# Integrative Bioinformatics Approaches Indicate a Particular Pattern of Some SARS-CoV-2 and Non-SARS-CoV-2 Proteins

**DOI:** 10.3390/vaccines11010038

**Published:** 2022-12-23

**Authors:** Chiranjib Chakraborty, Manojit Bhattacharya, Srijan Chatterjee, Ashish Ranjan Sharma, Rudra P. Saha, Kuldeep Dhama, Govindasamy Agoramoorthy

**Affiliations:** 1 Department of Biotechnology, School of Life Science and Biotechnology, Adamas University, Kolkata 700126, West Bengal, India; 2 Department of Zoology, Fakir Mohan University, Vyasa Vihar, Balasore 756020, Odisha, India; 3 Institute for Skeletal Aging and Orthopaedic Surgery, Hallym University-Chuncheon Sacred Heart Hospital, Chuncheon-si 24252, Gangwon-do, Republic of Korea; 4 Division of Pathology, ICAR-Indian Veterinary Research Institute, Izatnagar, Bareilly 243122, Uttar Pradesh, India; 5 College of Pharmacy and Health Care, Tajen University, Yanpu 907, Pingtung, Taiwan

**Keywords:** pattern recognition, protein-like alphabets, SARS-CoV-2 proteins, image comparison, structural symmetry

## Abstract

Pattern recognition plays a critical role in integrative bioinformatics to determine the structural patterns of proteins of viruses such as SARS-CoV-2. This study identifies the pattern of SARS-CoV-2 proteins to depict the structure–function relationships of the protein alphabets of SARS-CoV-2 and COVID-19. The assembly enumeration algorithm, Anisotropic Network Model, Gaussian Network Model, Markovian Stochastic Model, and image comparison protein-like alphabets were used. The distance score was the lowest with 22 for “I” and highest with 40 for “9”. For post-processing and decision, two protein alphabets “C” (PDB ID: 6XC3) and “S” (PDB ID: 7OYG) were evaluated to understand the structural, functional, and evolutionary relationships, and we found uniqueness in the functionality of proteins. Here, models were constructed using “SARS-CoV-2 proteins” (12 numbers) and “non-SARS-CoV-2 proteins” (14 numbers) to create two words, “SARS-CoV-2” and “COVID-19”. Similarly, we developed two slogans: “Vaccinate the world against COVID-19” and “Say no to SARS-CoV-2”, which were made with the proteins structure. It might generate vaccine-related interest to broad reader categories. Finally, the evolutionary process appears to enhance the protein structure smoothly to provide suitable functionality shaped by natural selection.

## 1. Introduction

**Figure 1:**
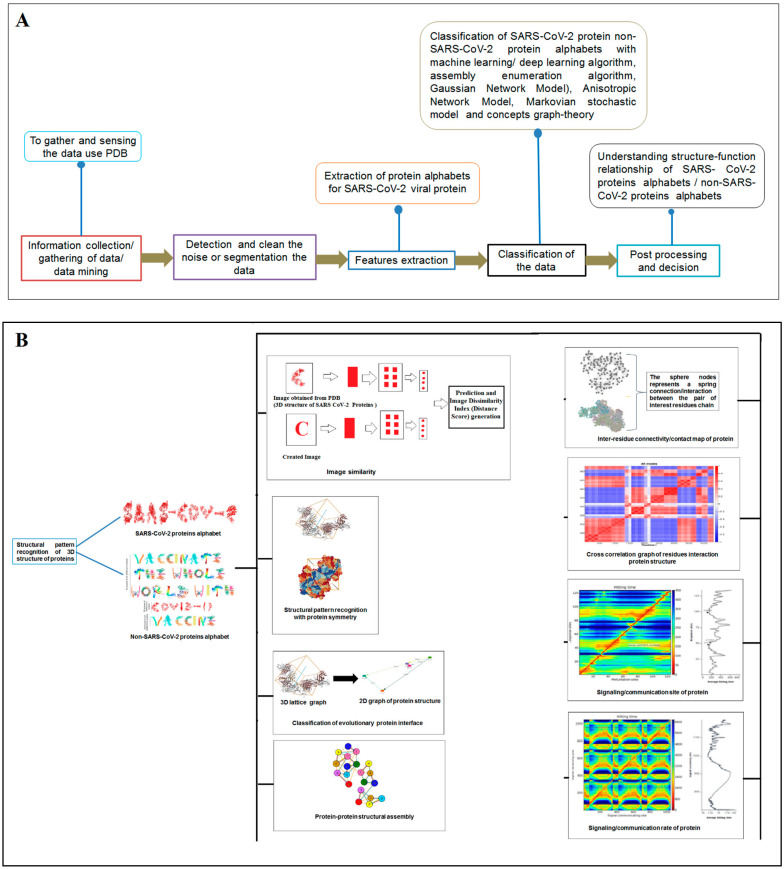

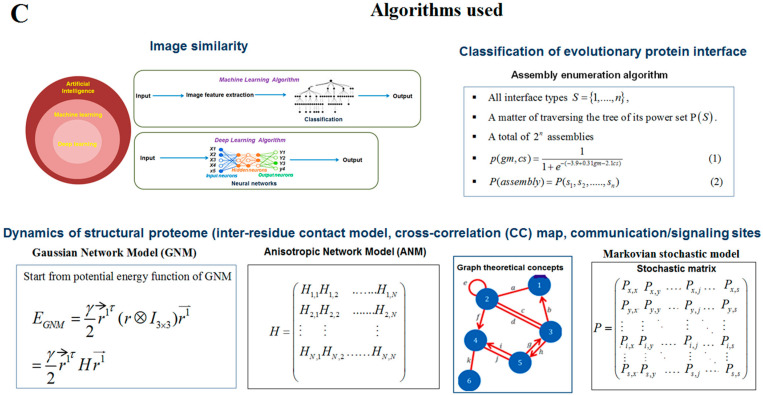
The schematic diagram shows the study and the applied algorithms in the study. (**A**) The flowchart shows the general steps of pattern recognition and our performed process. (**B**) Schematic diagram shows the different methods applied in this study. (**C**) The illustration shows the different algorithms involved in this study and their features.

Nature has created an enormous diversity of patterns in diverse life forms. To understand the archetype, pattern recognition has been used by scientists to depict the structural and prototype similarities, and only then can the classification from noisy data to distinguishable data be smoothly completed by using structural designs, statistical inputs, big data analytics, and image inspections computational investigations [1,2,3]. There are several steps involved in pattern recognition, which include information collection, data segmentation and classification, feature extraction, post-processing, and decision making (Figure 1A).

Pattern recognition is a predominant area in statistics, where scientists use data to develop theories, generate models, and apply methods for dimensional reduction, clustering, and classification through various approaches. Similarly, density estimation is one of the significant areas of statistical pattern recognition that uses normal-based models, normal mixture models, and Bayesian methods to estimate datasets for final application to recognize patterns [4,5].

In computer science, pattern recognition is essential, since scientists often apply big data and image analysis, plus computer graphics, to discover hidden messages [6,7]. However, pattern recognition is a challenging task because it is not easy to locate a rhythm from the noisy data. However, several algorithms are being applied in pattern recognition, which include clustering, machine learning, deep learning, multi-linear subspace learning, and deep learning [8,9,10,11,12,13,14,15].

Although pattern recognition in bio-science is complicated, reports showed that bioinformatics could play a significant role in solving the existing difficulties [16,17,18]. An example is pattern recognition in the structures of proteins as they form the foundation for life. Computational algorithms could help to predict the information from 3D structures by analyzing the naturally evolved proteins and their pattern similarities to better understand the evolutionary history [19,20].

Researchers have been trying to solve the structure of SARS-CoV-2 proteins ever since the start of the pandemic [21,22]. As of 8 September 2021, 1449 3D macro-molecular structural forms of SARS-CoV-2 have been solved and deposited in PDB. SARS-CoV-2 variants are developing due to diversified mutations [23,24,25,26,27,28,29]. During the SARS-CoV-2 mutation, the robustness and plasticity of the proteins change, which affects the overall functionality of the domains, especially the functional mechanisms of proteins [30]. To understand the evolution of viral variants, protein information fetching is essential. Therefore, it is critical to recognize the pattern in SARS-CoV-2 protein 3D structure models using different algorithms to explore the evolutionary clues to the rapidly evolving variants.

Understanding the structural symmetry of elements is a significant process of pattern recognition [31,32]. Scientists are investigating the symmetry in protein architecture [33,34,35], since it determines how proteins interact with each other. The structural basis of the capsid in HIV-1 is an essential factor to be recognized by the host proteins CPSF6 [36]. Together, a structural symmetry needs to be identified to infer the functionality and understanding of the driving forces of evolution [37,38]. Using a web platform, researchers can analyze and visualize the structure of a protein. In this direction, a recent web app, called Mol* Viewer, hosted on GitHub, provides structural symmetry. The app can be used to understand pattern recognition [39]. Another robust algorithm is the evolutionary protein interface classifier (EPPIC), which merges the two results of an interface classification, which includes topology and symmetry. This algorithm is represented through a catalogue of assemblies of the inner crystal structure coordinates. Then, the algorithm generates probabilistic scores from an evolutionary scoring system, called pairwise scoring, from the most likely assembly. The classifier of evolutionary protein interface is the best among the two necessary signatures, namely pairwise interface classification and the assembly enumeration algorithm [40]. The protein classifier classifies the interface SARS-CoV-2 proteins 3D structure as alphabets for structural pattern recognition.

Scientists extract data from different proteins 3D structures using algorithms, such as deep learning and machine learning [41,42]. Therefore, it is necessary to solve the dynamics of structurally resolved protein structures to better understand the pattern recognition. Several models have recently been generated using a network model interface that uses the Gaussian network model (GNM) and anisotropic network model (ANM). The interface can construct different protein structure–function models, such as inter-residue contact signature, fluctuations of cross-correlations between residue, communication/signaling sites of protein for intramolecular communication, etc. They can be used to identify patterns [43]. A model for the signal communication of a protein can be generated through hitting times and commute times/sites by using the concepts of graph theory and the Markovian stochastic model [44]. Proteome and its structural dynamics can be analyzed through the DynOmics computational interface. With the model, similar to the inter-residue contact model, fluctuations of cross-correlations between the residue and communication/signaling sites of protein can be generated through this computational interface. However, the pattern of any SARS-CoV-2 proteins 3D structure and their structure–function relationship can be understood with the help of structural symmetry, evolutionary protein classification, the dynamics of the structural proteome, etc. In this direction, pattern recognition with 3D structure of SARS-CoV-2 proteins was performed (Figure 1B). For analyzing the 3D structure of SARS-CoV-2 proteins or non-SARS-CoV-2 proteins, several algorithms were used namely Deep AI model, assembly enumeration, anisotropic network model, Gaussian network model, and Markovian stochastic model (Figure 1C). The pattern identification was performed with the collected proteins alphabets from SARS-CoV-2 proteins and non-SARS-CoV-2 proteins. Image comparison was performed using protein-like alphabets with English alphabets. The structural symmetry pattern, evolutionary protein classification, and structural proteome dynamics were also considered. Inter-residue contacts and developed inter-residue contact models (both residue and chain) were created to illustrate cross-correlations between residues through a cross-correlation (CC) map. To understand the functionality, the communication/signaling sites of protein residue and signal communication/signal receiving rate of protein alphabet were analysed, which led to the creation of a structural functioning relationship of the SARS-CoV-2 proteins.

## 2. Materials and Methods

### 2.1. Data Mining Using PDB and Collection of Proteins as Alphabets from SARS-CoV-2 Proteins and Non-SARS-CoV-2 Proteins

A pattern was discovered in the 3D structures of SARS-CoV-2 proteins. The Protein Data Bank (PDB) was extensively used to retrieve alphabets, such as patterns from SARS-CoV-2 proteins, to design of various 3D structures of SARS-CoV-2 proteins [45].

For developing two slogans for our paper, first, we tried to find similarities in the structural pattern of some SARS-CoV-2 proteins with the English alphabet. Some are not found in the SARS-CoV-2 proteins. In this case, some “non- SARS-CoV-2 proteins” were selected, similar to the English alphabet. We have added this part in the method section of the manuscript.

Images of general alphabets were created and compared for similarities between the protein alphabets and English alphabets by using the image similarity API (application programming interface) [46]. The image similarity API developed a distance score, as similarity index/dissimilarity index.

Again, we selected four protein alphabets with antibodies/immunological or vaccine-associated roles collected from protein alphabets pools. Distance score was also developed as protein alphabets to understand the similarity index/dissimilarity index.

### 2.2. Pattern Recognition of 3D Structures of SARS-CoV-2 and Non-SARS-CoV-2 Proteins

Structural pattern recognition of 3D structures of SARS-CoV-2 proteins was analyzed to understand the variations and patterns in symmetry. The pattern was evaluated using Mol* Viewer, a recent web app and modern software that provides structural symmetry of a protein. The Mol* Viewer was used to understand the structural pattern recognition [38].

### 2.3. Pattern Recognition Using the Classification of Evolutionary Protein Interface through Assembly Enumeration Algorithm

Computational interface was used to generate 2D graph of SARS-CoV-2 proteins 3D structural alphabets. We used an evolutionary protein interface classifier (EPPIC) to evaluate the assemblies inside the crystal structure coordinates. Using the assembly enumeration algorithm, the interface evaluated 3D structure (input PDB files) and generated a 3D lattice graph of the protein’s crystal structure. Then, it generated 2D graph of the protein assembly [39].

### 2.4. Pattern Recognition Using the Protein–Protein Interface of 3D Structures of SARS-CoV-2 and Non-SARS-CoV-2

The interface of 3D structures of a protein is essential for their function. The pattern recognition of protein structural assembly was studied using the protein–protein interface of 3D structures of SARS-CoV-2 proteins and non-SARS-CoV-2 proteins. PDBSum was used to study a protein–protein interface of the 3D structure of a protein [47,48].

### 2.5. Pattern Recognition with Dynamics of Structural Proteome

First, two types of inter-residue contact models were created, which include the usage of atoms and the usage of chains. Then, DynOmics computational interface contact model was used to measure fluctuations of cross-correlations between residue and communication/signaling sites of protein [40]. From the changes of cross-correlations between residues, a cross-correlation (CC) map was generated. During the generation of the CC map, the interface calculated residue numbers in (i,j) alongside the axes. Communication/signaling sites of protein were analyzed through signal communication/signal receiving efficiency, signal communication/signal receiving rate, and stand deviation of hitting time. All the maps were generated through the calculated residue numbers in (i,j) alongside the axes.

### 2.6. Post-Processing and Decision

Finally, to evaluate the patterns generated from the collection of protein alphabets, especially using SARS-CoV-2 proteins, structural symmetry, classification of evolutionary protein interface of protein alphabets, and the dynamics of the structural proteome of protein alphabets were processed. The final correlation of structure–function relationship of SARS-CoV-2 proteins was created at last.

## 3. Result

### 3.1. Data Mining Using PDB and Collection of Proteins as Alphabets from SARS-CoV-2 and Non-SARS-CoV-2 Proteins

**Figure 2:**
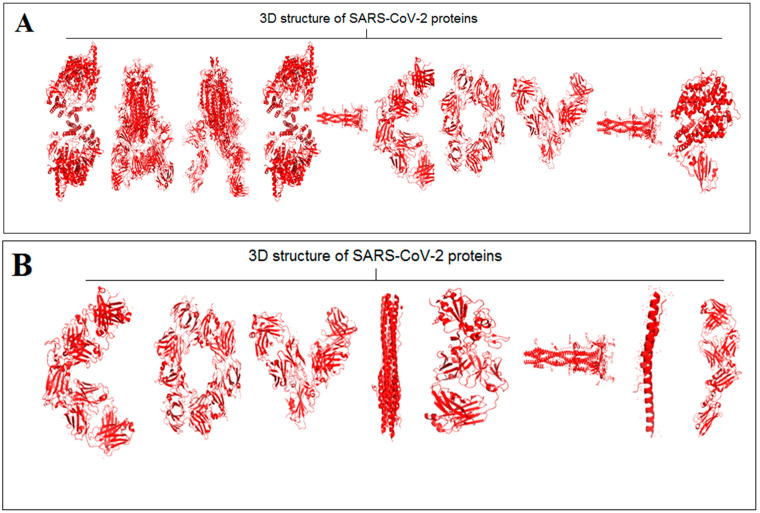

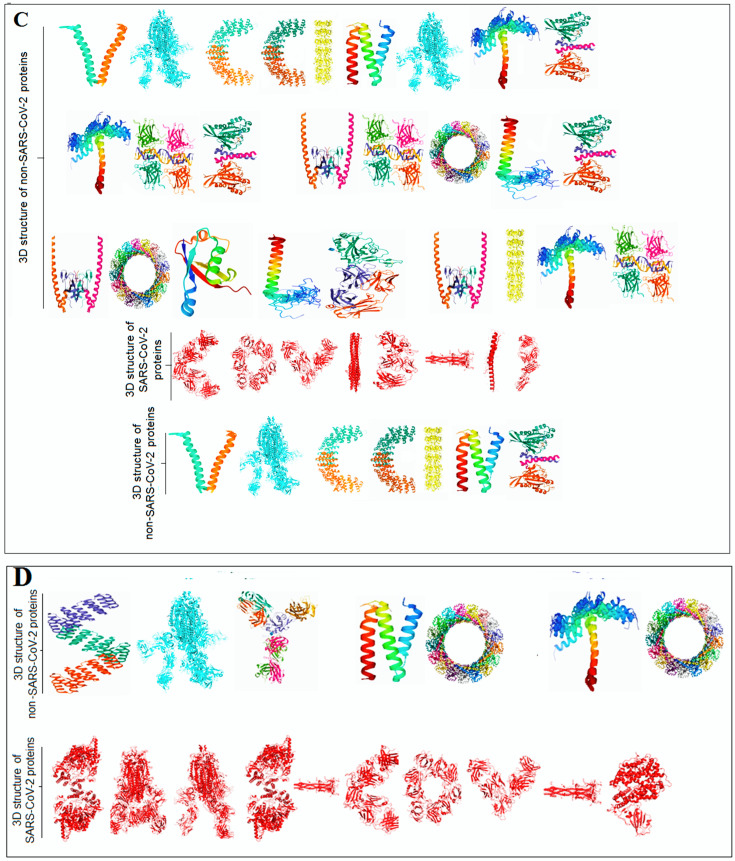

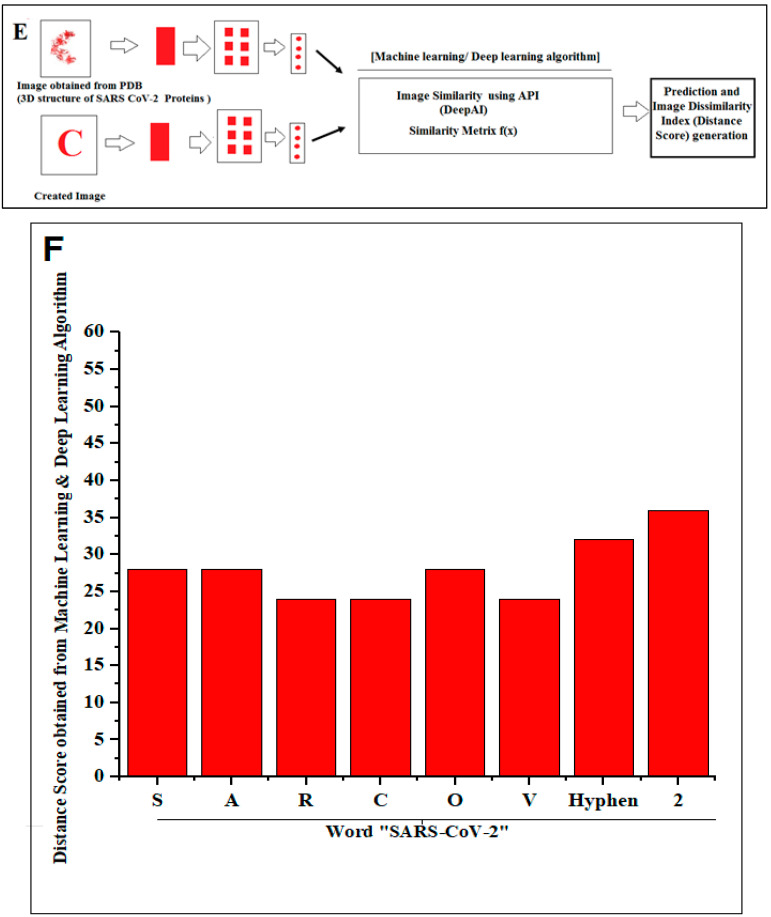

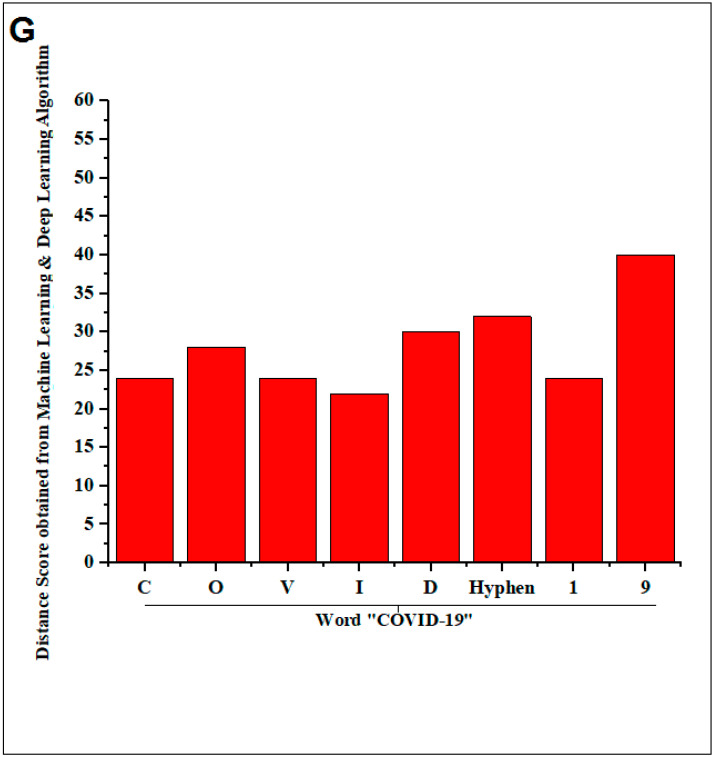

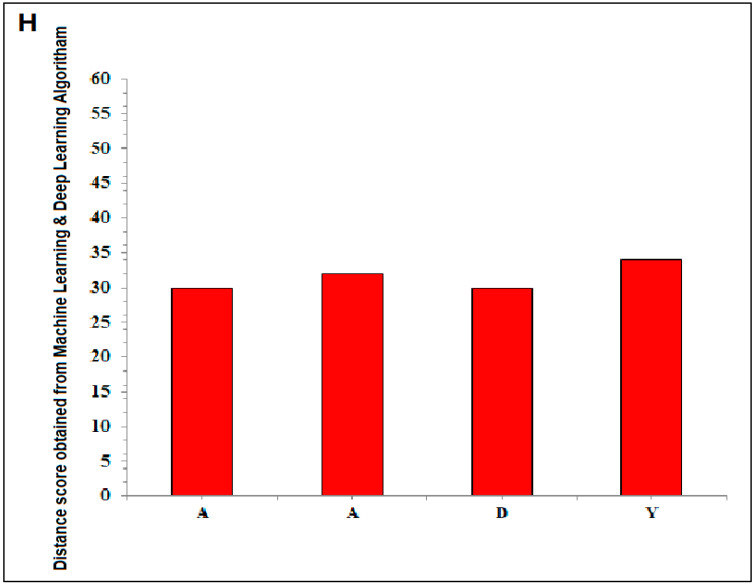
The representation shows the created two words with SARS-CoV-2 proteins and two slogans with non-SARS CoV-2 proteins. (**A**) The diagram shows the created word of “SARS-CoV-2” using protein alphabets. (**B**) The diagram shows the created word of “COVID-19,” using protein alphabets. (**C**) The diagram shows the developed first slogan, “VACCINATE THE WHOLE WORLD WITH COVID-19 VACCINE,” using protein alphabets. (**D**) The diagram shows the developed second slogan, “SAY NO TO SARS-CoV-2,” using protein alphabets. (**E**) The schematic diagram shows the process of image comparison and distance score generation. (**F**) Graphical representation of the generated distance score of each protein alphabet of “SARS-CoV-2”. (**G**) Graphical representation of the generated distance score of each protein alphabet of “COVID-19”. (**H**) Graphical representation of the generated distance score of each protein alphabet of four SARS-CoV-2 protein alphabets with antibodies/immunological or vaccine-associated roles. The analysis tried to create the pattern of protein structure and compute the image similarity/dissimilarity of 3D structures of each protein.

Extraction of protein alphabets from SARS-CoV-2 proteins was performed to develop two words, i.e., “SARS-CoV-2” (Figure 2A) and “COVID-19” (Figure 2B). In order to create the words “SARS-CoV-2” and “COVID-19”, the SARS-CoV-2 protein structural patterns as letters were recorded with their PDB ID, as noted in Tables S1 and S2, respectively. Using the nature-created SARS-CoV-2 proteins alphabets, “SARS-CoV-2” and “COVID-19” words with red colors were created to provide color effects on the two words as danger indications.

Two slogans using the diversified 3D structures of proteins as alphabets were included. The PDB was searched extensively to derive different protein alphabets from SARS-CoV-2 and non-SARS-CoV-2. Finally, two slogans were generated using the protein alphabets: the first was titled, “VACCINATE THE WHOLE WORLD WITH COVID-19 VACCINE” (Figure 2C), and all proteins as alphabets and their PDB IDs are noted in Table S3; the second was titled, “SAY NO TO SARS-CoV-2” (Figure 2D), and all proteins as alphabets and their PDB IDs are as noted in Table S4.

This study fetched the 12-number SARS-CoV-2 proteins and the 14-number non-SARS-CoV-2 proteins to design the words and slogans. Again, the 12-number SARS-CoV-2 proteins alphabet was compared with the English alphabets, and a distance score was generated after image comparison. The lists between the protein alphabets and English alphabets for “SARS-CoV-2” and “COVID-19” are recorded in Tables S5 and S6, respectively. The concept of distance score generation is shown in Figure 2E. The distance score generated from each alphabet of “SARS-CoV-2” and “COVID-19” is recorded in Figure 2F,G. After image comparison, the distance score of “I” was observed as the lowest distance score, which was 22. At the same time, the distance score of “9” was noted as the highest distance score, which was 40.

The study also fetched four SARS-CoV-2 protein alphabets with antibodies/immunological or vaccine-associated roles from our previous protein alphabets pools. A detailed description of these alphabets with the PDB id is recorded in Table 1. For the image comparison, the generated alphabets and the protein alphabets used in the image comparison study were recorded in Table S7. The distance score generated using four SARS-CoV-2 protein alphabets with antibodies/immunological or vaccine-associated roles were recorded in Figure 2H. In this case, “A” was the lowest distance score, with 30. At the same time, the distance score of “Y” was the highest distance score, which was 34.

### 3.2. Structural Pattern Recognition of 3D Structures of SARS-CoV-2 and Non-SARS-CoV-2 Proteins

**Figure 3:**
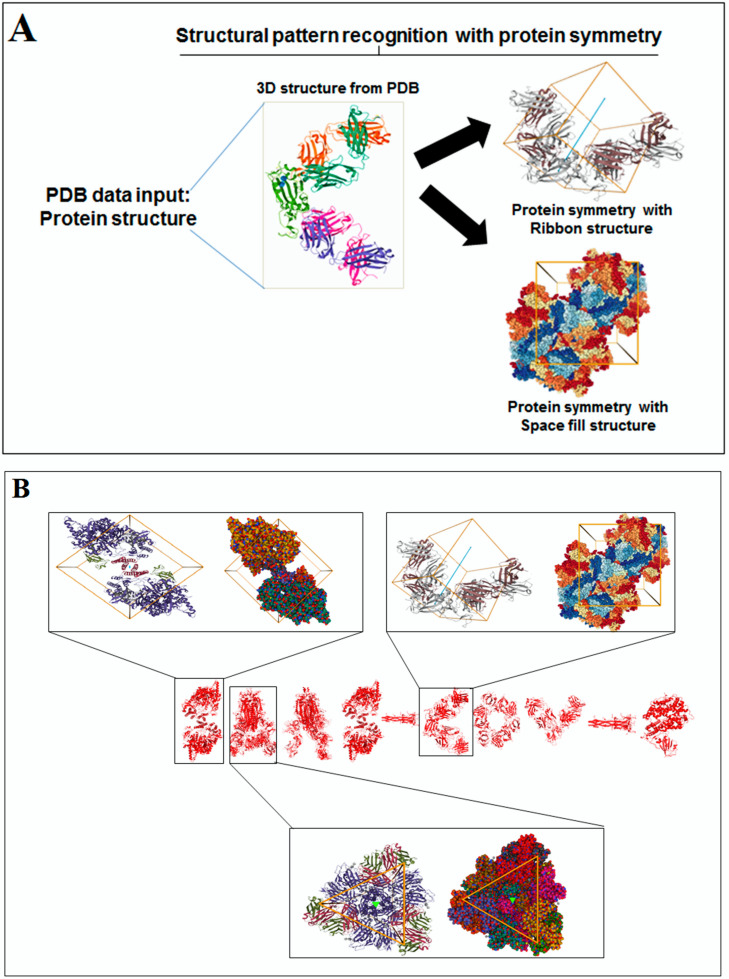

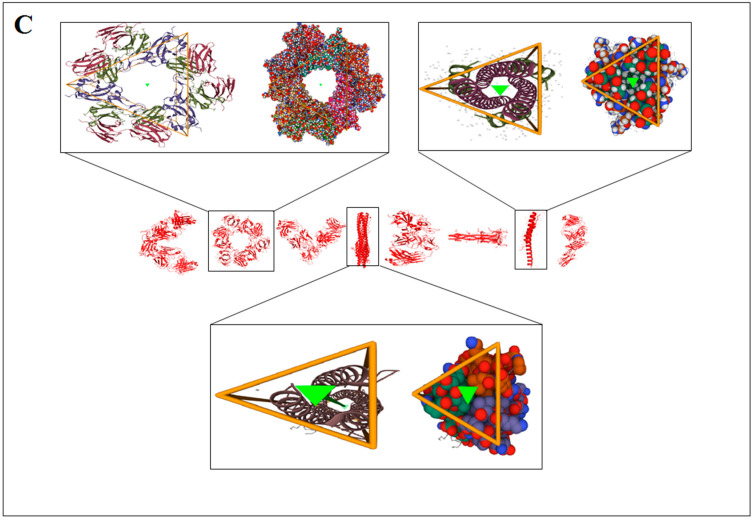

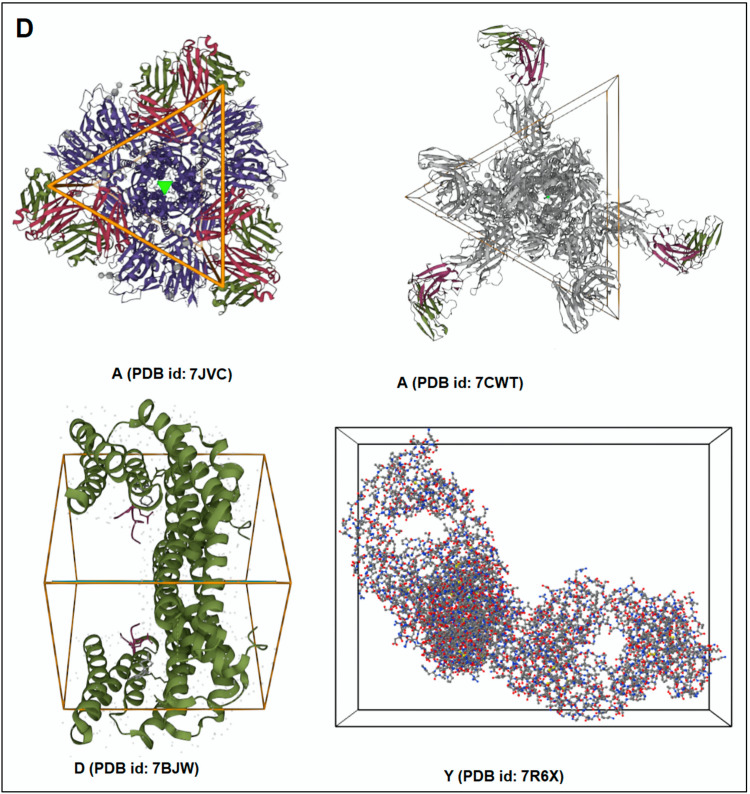
The schematic representation structural pattern analysis and the result the study of protein alphabets of SARS-CoV-2 proteins and non-SARS-CoV-2 proteins. (**A**) schematic representation of structural pattern evaluation study. (**B**) Structural symmetry of each protein, which was used to develop the word “SARS-CoV-2”. (**C**) Structural symmetry of each protein, which was used to develop the word “COVID-19”. (**D**) Structural symmetry of four SARS-CoV-2 protein alphabets with antibodies/immunological or vaccine-associated roles. The study tried to analyze the structural pattern recognition through structural symmetry of 3D structures of each protein.

The concept of structural pattern recognition to understand the structural symmetry pattern is shown in Figure 3A. Structural pattern recognition of protein alphabets of “SARS-CoV-2” and their structural symmetry pattern are indicated in Figure 3B. At the same time, structural pattern recognition of protein alphabets of “COVID-19” and their structural symmetry pattern are noted in Figure 3C. Similarly, structural pattern recognition of four SARS-CoV-2 protein alphabets with antibodies/immunological or vaccine-associated roles” and their structural symmetry pattern are noted in Figure 3D.

A structural symmetry pattern of protein alphabets “SARS-CoV-2” and “COVID-19”, was developed with the letters C, O, I, Hyphen(-), 1(One), S, and A. At the same time, non-symmetric proteins were also found from “SARS-CoV-2” and “COVID-19”, which were V, D, 9, R, and 2. From the generated two slogans, symmetric proteins for the rest of the words other than COVID-19 and SARS-CoV-2 were V, A, C, I, E, W, H, and O. Similarly, non-symmetric proteins from the words other than COVID-19 and SARS-CoV-2 were N, L, S, Y, and R.

At the same time, the symmetrical structure of the non-SARS-CoV-2 proteins was also illustrated in Figure S1.

### 3.3. Pattern Recognition Using the Classification of Evolutionary Protein Interface through Assembly Enumeration Algorithm

**Figure 4:**
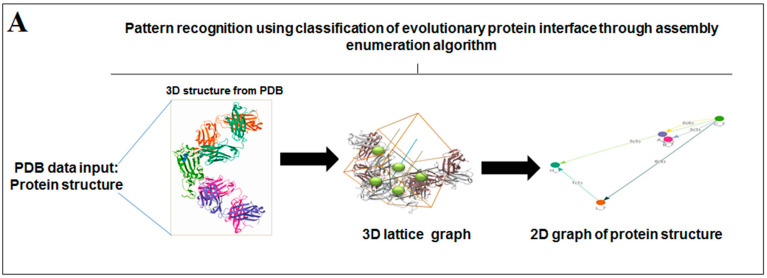

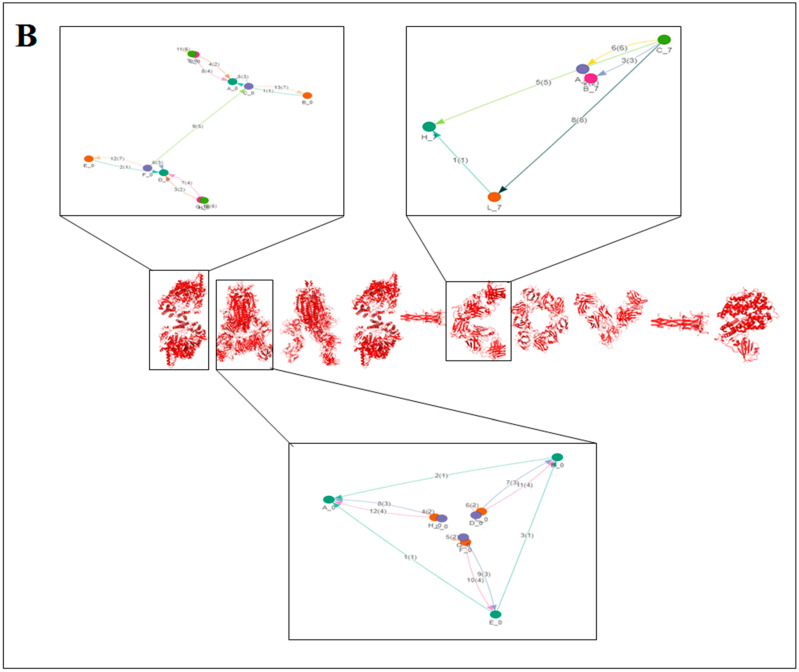

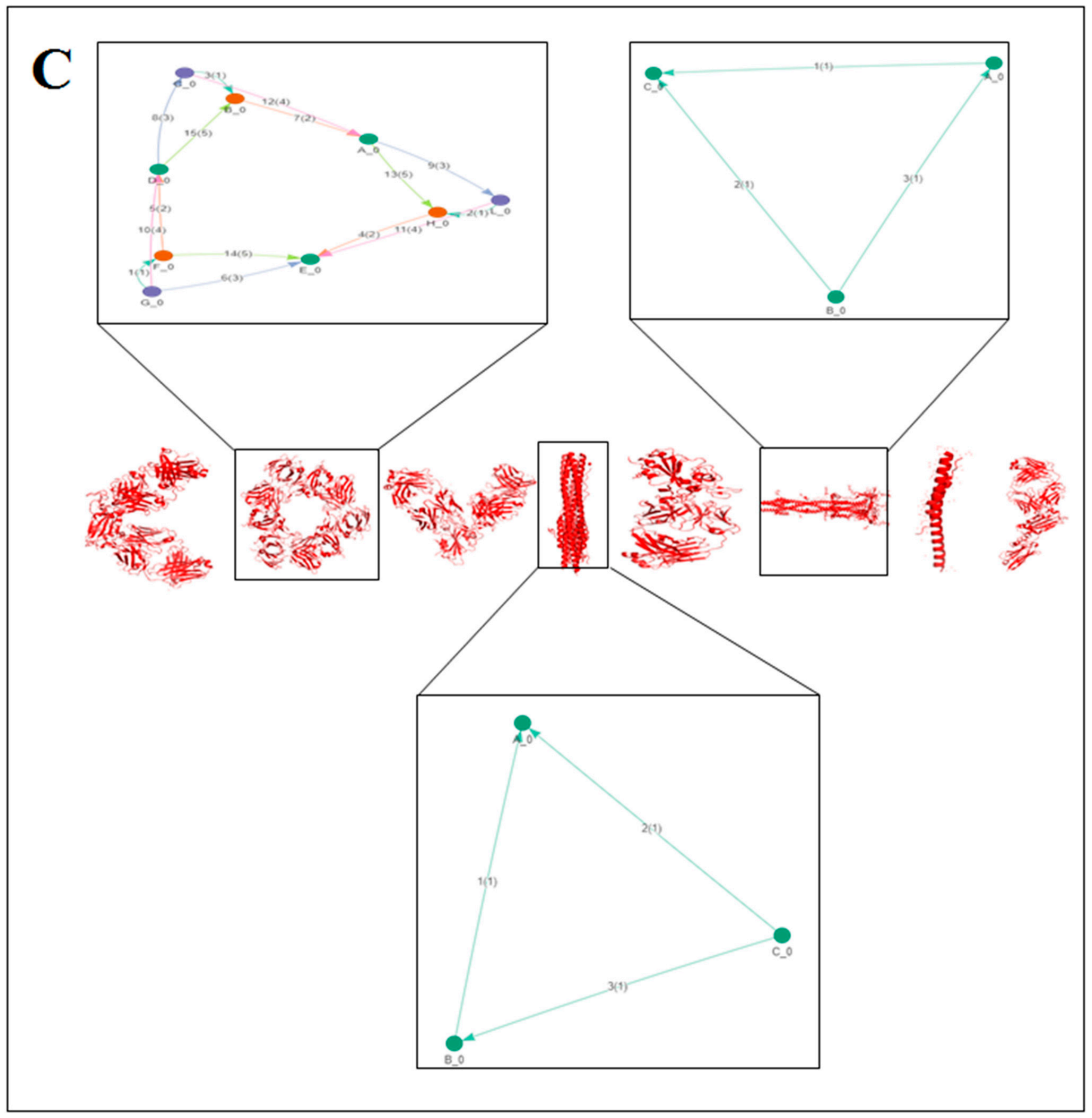

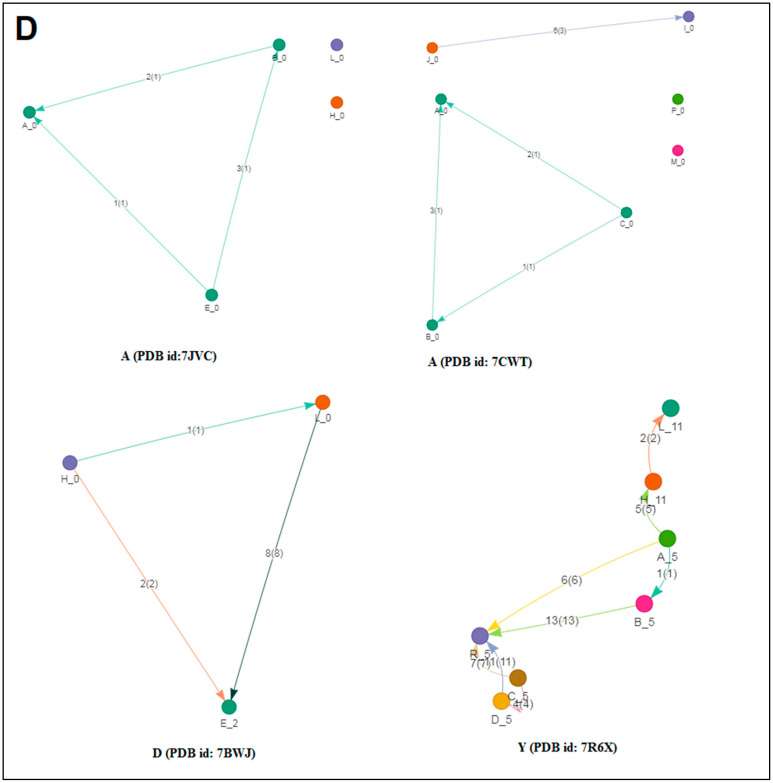
The schematic representation on the classification of evolutionary protein interface and the result of the study of protein alphabets of SARS-CoV-2 proteins and non-SARS-CoV-2 proteins. (**A**) Schematic representation of the classification of evolutionary protein interface study. (**B**) Classification of evolutionary protein interface of each protein, which was used to develop the word “SARS-CoV-2”. (**C**) Classification of evolutionary protein interface of each protein, which was used to develop the word “COVID-19”. (**D**) Classification of evolutionary protein interface of four SARS-CoV-2 protein alphabets with antibodies/immunological or vaccine-associated roles used in this study. The study analyzed the structural pattern of 3D structures of each protein through the classification of evolutionary protein interface.

The classification of evolutionary protein interface is shown in Figure 4A. A lattice graph was created to represent an in-depth architecture of the mathematical representation of crystal nets.

The classification of the evolutionary protein interface of protein alphabets of “SARS-CoV-2” and their lattice graph in the 2D representation of the protein assembly are shown in Figure 4B. In unison, the classification of the evolutionary protein interface of protein alphabets of “COVID-19” and their lattice graph in the 2D representation of the protein assembly are also shown in Figure 4C. At the same time, the classification of the evolutionary protein interfaces of four SARS-CoV-2 protein alphabets with antibodies/immunological or vaccine-associated roles and their lattice graph in the 2D representation of the protein assembly are also shown in Figure 4D. Similarly, the study also depicts the classification of evolutionary protein interface of all non-SARS-CoV-2 proteins, which are displayed in Figure S2.

### 3.4. Pattern Recognition Using Protein–Protein Interface 3D Structures of SARS-CoV-2 and Non-SARS-CoV-2

**Figure 5:**
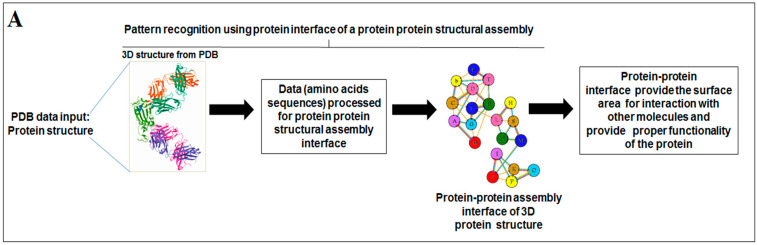

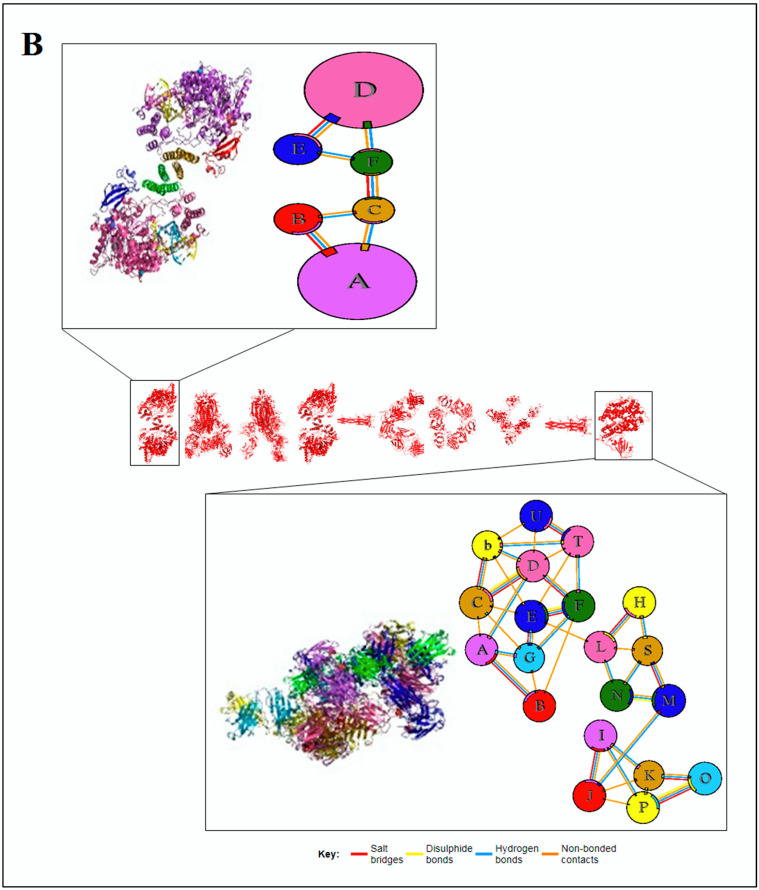

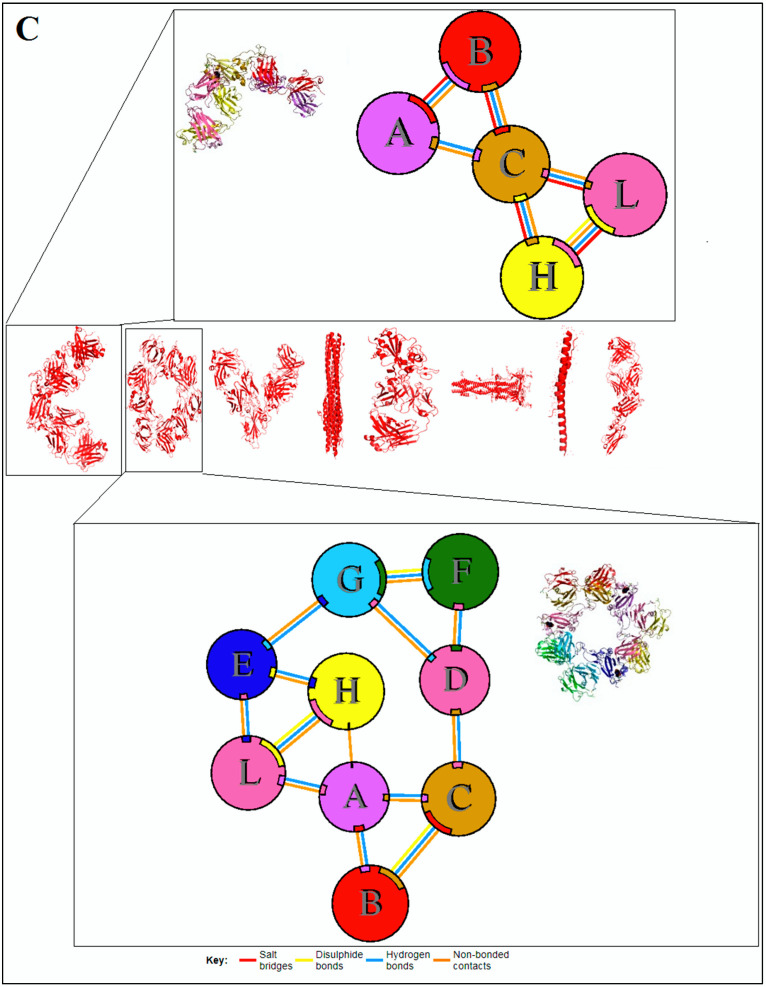

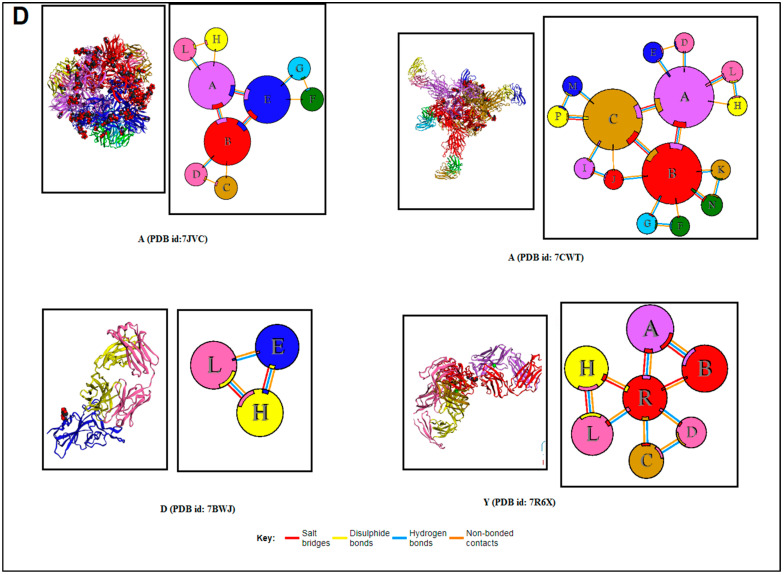
The schematic representation and study outcome of pattern recognition using protein–protein interface, inter-residue contact model, spring interaction/connection between the pair of interest nodes or chains and to develop a cross-correlation (CC) map, 2D map of communication/signaling sites and hitting/signal communication times, 2D map for the signaling rate, signaling receiving time, and signaling communication time of the SARS-CoV-2 proteins and non-SARS-CoV-2 proteins. (**A**) Schematic representation of pattern recognition using protein–protein interface 3D structures. (**B**) protein–protein interface 3D structures of each protein, which were used to develop the word “SARS-CoV-2”. (**C**) The protein–protein interface 3D structures of each protein, which were used to create the word “COVID-19”. (**D**) The protein–protein interface 3D structures of our SARS-CoV-2 protein alphabets with antibodies/immunological or vaccine-associated roles used in this study. The study (Figure 5A–D) tried to analyze the structural pattern of 3D forms of each protein and their assemblies through the protein–protein interface.

The interface of the assemblies is essential for understanding the clues of the pattern of the 3D protein structure; therefore, this study focused on the interface of protein chain assemblies. The protein chain assemblies provide the proper shape and surface area of the protein to give appropriate functionality, as shown in the schematic diagram depicted in Figure 5A. We studied the pattern using the protein–protein interface of 3D structures of proteins alphabets, which were used to build the word ‘SARS-CoV-2′ (Figure 5B). At the same time, we evaluated protein–protein interface of 3D structures of proteins alphabets used to build the word ‘COVID-19′ (Figure 5C). Finally, we evaluated the protein–protein interface of 3D structures of four SARS-CoV-2 protein alphabets with antibodies/immunological or vaccine-associated roles (Figure 5D).

Similarly, our analyses evaluated the pattern of the protein–protein interface of 3D structures of non-SARS-CoV-2 proteins alphabets, which are displayed in Figure S3.

### 3.5. Pattern Recognition with Dynamics of Structural Proteome

**Figure 6:**
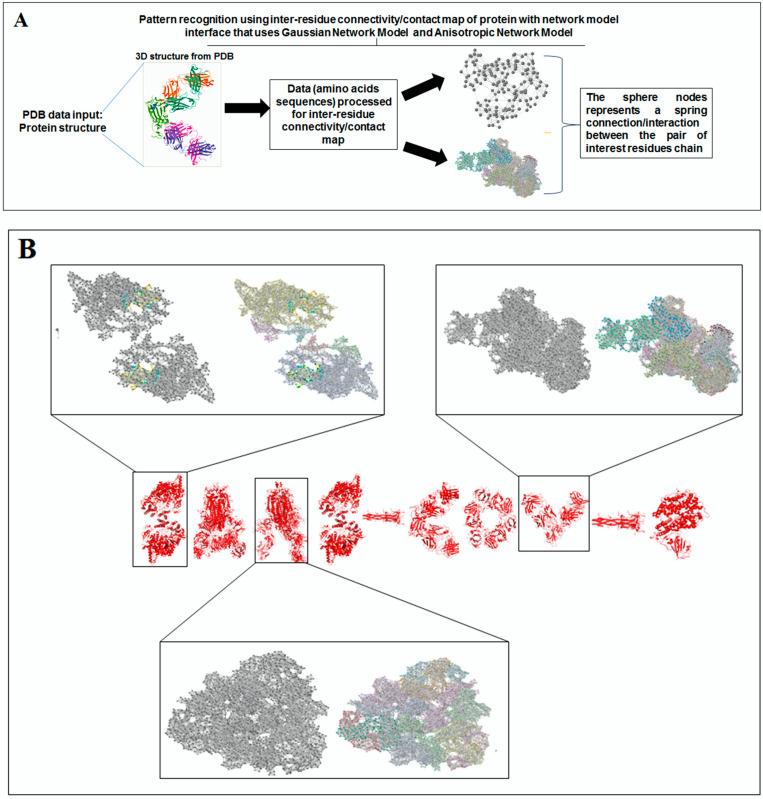

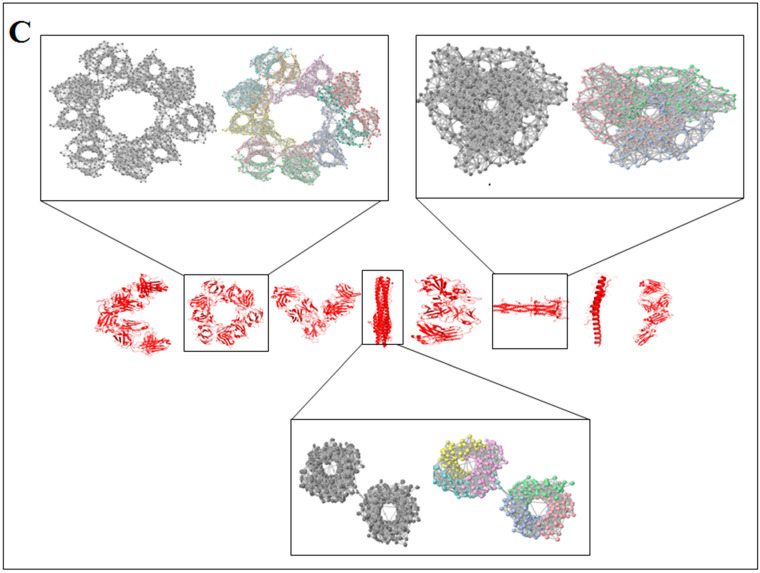

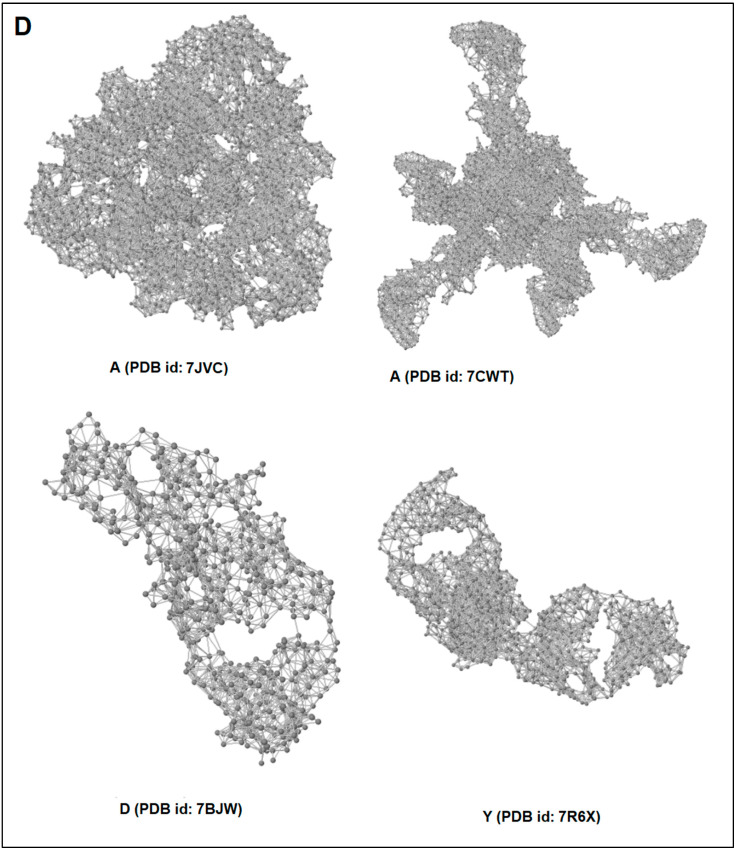
The schematic representation shows the inter-residue contact model and the outcome of the study of SARS-CoV-2 proteins and non-SARS-CoV-2 proteins. (**A**) Schematic representation of inter-residue contact model. (**B**) Inter-residue contact model of all residues and chains of 3D structures of proteins, which were used to develop the word “SARS-CoV-2”. (**C**) Inter-residue contact model of all residues and chains of 3D structures of proteins, which were used to create the word “COVID-19”. (**D**) Inter-residue contact model of all residues of 3D structures of our SARS-CoV-2 protein alphabets with antibodies/immunological or vaccine-associated roles. The study analyzed the structural pattern of 3D structures of each protein of the residues of each protein through an inter-residue contact model of all residues and chains.

To understand the dynamics of the structural proteome, we created an inter-residue contact model representing through the nodes in a 3D protein, which provides the landscape of a spring connection or interaction between the pair of interest residues or chains (Figure 6A). Different nodes symbolize a spring interaction/relationship between the interest residues or chains. We have depicted two forms of inter-residue contact models: the first one is for all residues involved in the interaction. The second is for all chains involved in the interaction. These two models were built with the 3D structures of the protein alphabet involved in developing the word ‘SARS-CoV-2′ (Figure 6B). These two models were created using 3D structures of the protein alphabet engaging in developing the word “COVID-19′’ (Figure 6C). Again, we developed inter-residue contact models of the evolutionary protein interface of four SARS-CoV-2 protein alphabets with antibodies/immunological or vaccine-associated roles (Figure 6D). In this case, we developed the first model. Likewise, the study evaluated the inter-residue contact model for all residues and chains of 3D structures of non-SARS-CoV-2 proteins alphabets, as displayed in Figure S4.

**Figure 7:**
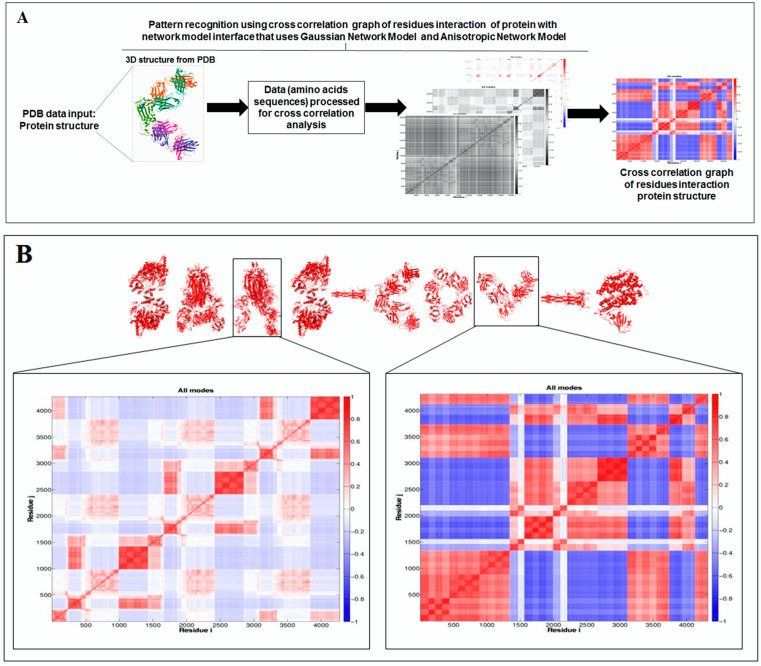

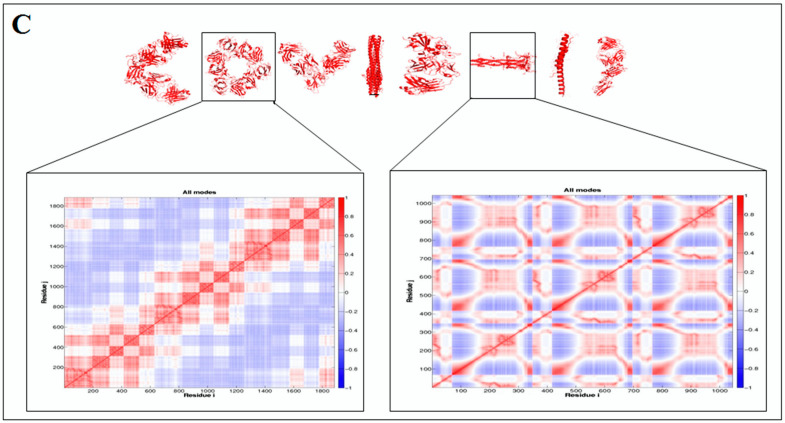

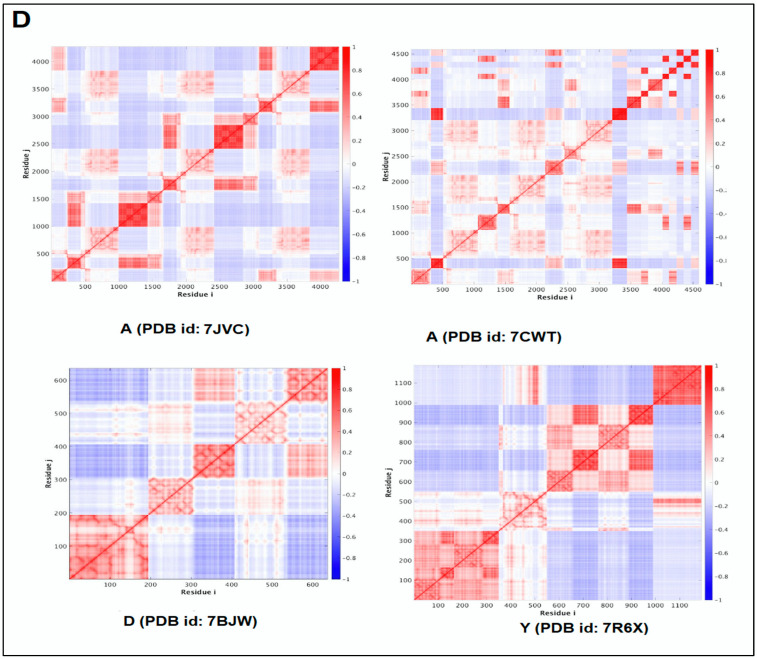
The schematic representation shows the spring interaction/connection between the pair of interest nodes or chains and to develop a cross-correlation (CC) map from the study of SARS-CoV-2 proteins and non-SARS CoV-2 proteins. (**A**) Schematic representation of spring interaction/connection between the pair of interest nodes or chains and to develop a cross-correlation (CC) map. (**B**) A cross-correlation (CC) map of 3D structures of proteins, which were used to develop the word “SARS-CoV-2”. (**C**) A cross-correlation (CC) map of all residues and chains of 3D structures of proteins, which were used to develop the word “COVID-19”. (**D**) A cross-correlation (CC) map of all residues and chains of 3D structures of four SARS-CoV-2 protein alphabets with antibodies/immunological or vaccine-associated roles used in this study. The study analyzed the structural pattern of 3D structures of each protein using spring interaction/connection between the pair of interest nodes or chains and to develop a cross-correlation (CC) map.

Furthermore, to understand the additional information about the dynamics of the structural proteome, we developed a cross-correlation (CC) map. The CC map provides extra information about the residue interaction pattern and residue fluctuations of a protein. However, the concept of the generation of the CC map is visualized in Figure 7A. Simultaneously, the CC map of protein alphabets of ‘SARS-CoV-2′ and “COVID-19” are represented in Figure 7B,C, respectively. Similarly, the CC maps of protein alphabets of four SARS-CoV-2 protein alphabets with antibodies/immunological or vaccine-associated roles were generated and depicted in Figure 7D.

Simultaneously, the study developed the CC map of 3D structures of non-SARS-CoV-2 proteins alphabets, as shown in Figure S5.

The cross-correlation (CC) map shows the calculated residue interface visualized (i,j) alongside the axes in the map.

**Figure 8:**
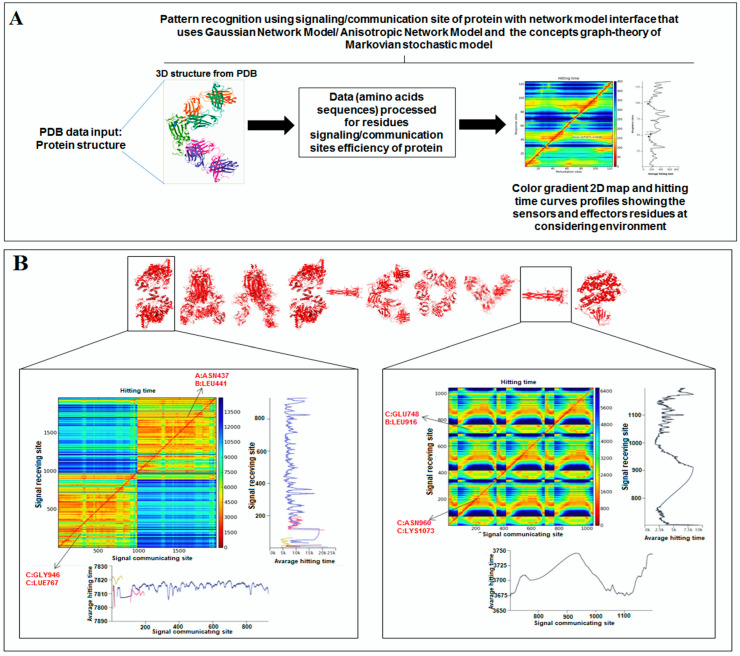

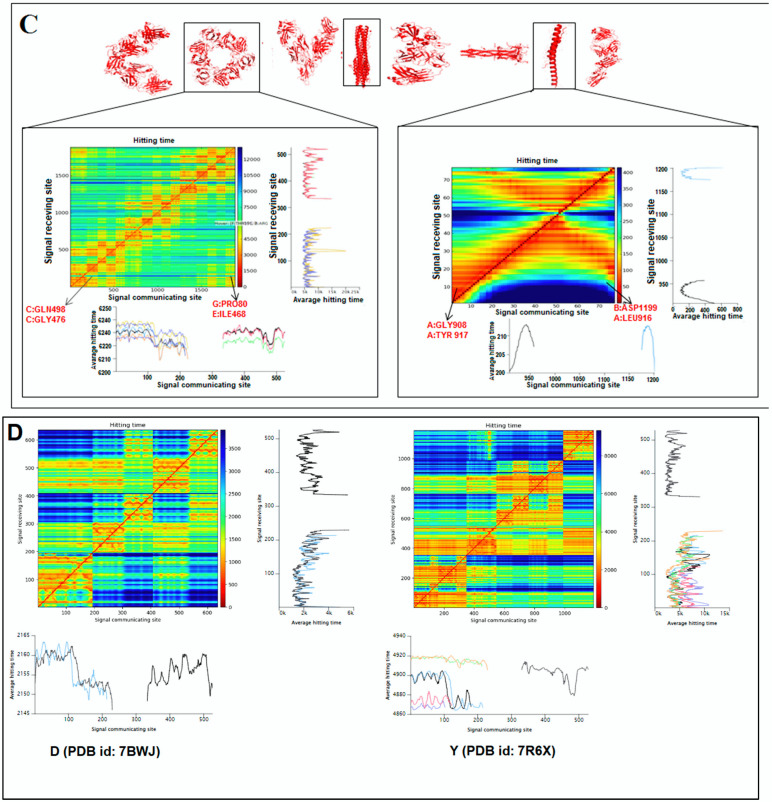
The schematic representation for the development of a 2D map of communication/signaling sites and hitting/signal communication times generated with protein residues using SARS-CoV-2 proteins and non-SARS-CoV-2 proteins. (**A**) The schematic representation for the development of a 2D map of communication/signaling sites and hitting/signal communication times generated with protein residues. (**B**) The 2D map for communication/signaling sites and hitting/signal communication times generated with protein residues of 3D structures of proteins which are used to develop the word “SARS CoV-2”. (**C**) The 2D map for communication/signaling sites and hitting/signal communication times generated with protein residues of 3D structures of proteins which are used to develop the word “COVID-19”. (**D**) The 2D map for communication/signaling sites and hitting/signal communication times generated with protein residues of 3D structures of two SARS-CoV-2 protein alphabets with antibodies/immunological or vaccine-associated roles used in this study. The complete analysis tried to capture the functionality of residue.

Using the hitting and signal communication times, researchers can generate protein residues from communication/signaling sites related to the residue’s functionality. The functional tendency of residues can be reflected in the map. It represents sending signal tendency, or to receive the trend of signals. The higher direction for communication can be indicated by the smaller hitting time (Figure 8A). The map also shows the perturbation site. The hitting and signal communication times/site of protein residues of ‘SARS-CoV-2′ and ‘COVID-19′ protein alphabets are represented in Figure 8B,C, respectively.

Finally, the hitting and signal communication times/site of protein alphabets of two SARS-CoV-2 protein alphabets with antibodies/immunological or vaccine-associated roles (“D” and “Y”) were generated and represented in Figure 8D.

The 2D maps were generated for communication/signaling sites and hitting/signal communication times using 3D structures of non-SARS-CoV-2 proteins, which were used to develop the two slogans (Figure S6).

**Figure 9:**
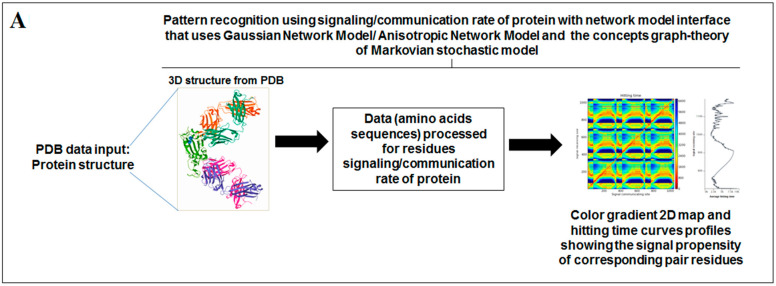

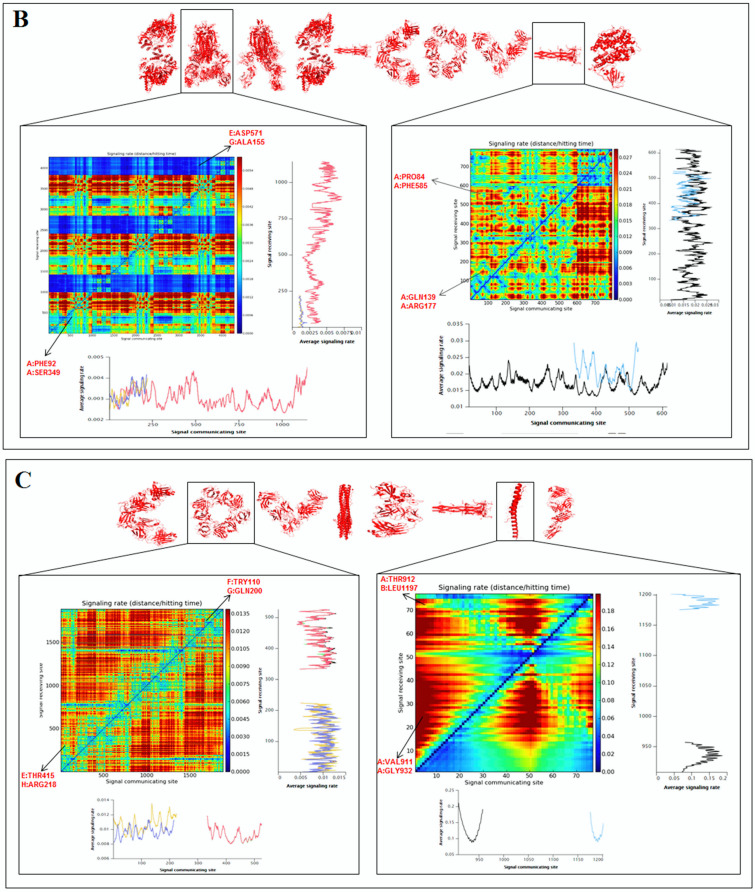

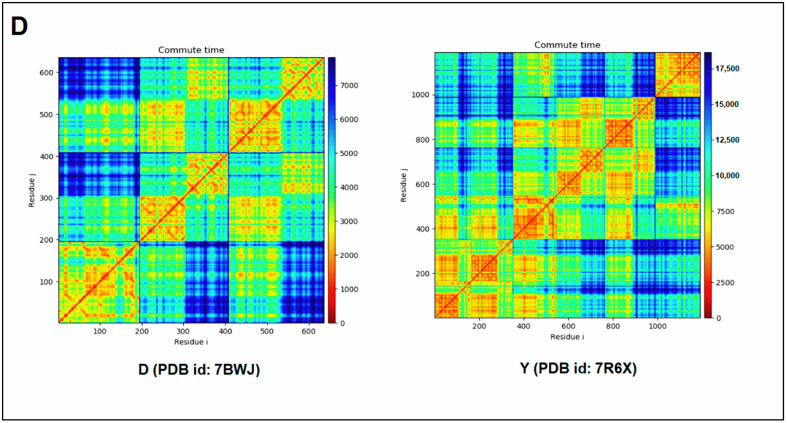
The schematic representation for developing a 2D map for the signaling rate, signaling receiving time, and signaling communication time from protein residues of SARS-CoV-2 proteins and non-SARS-CoV-2 proteins. (**A**) The schematic representation to develop a 2D map of the signaling rate, signaling receiving time, and signaling communication time from protein residues of SARS-CoV-2 proteins and non-SARS-CoV-2 proteins. (**B**) The 2D map of the signaling rate, signaling receiving time, and signaling communication time from protein residues of 3D structures of proteins which are used to develop the word “SARS-CoV-2”. (**C**) The 2D map of the signaling rate, signaling receiving time, and signaling communication time from protein residues of 3D structures of proteins which are used to develop the word “COVID-19”. (**D**) The 2D map of the signaling rate, signaling receiving time, and signaling communication time from protein residues of 3D structures of two SARS-CoV-2 protein alphabets with antibodies/immunological or vaccine-associated roles used in this study. The comprehensive analysis tried to capture the functionality of residue.

A color gradient 2D map was generated from the signaling rate, signaling receiving time, and signaling communication time from protein residues (Figure 9A). It also represents the functionality of the residue. The signaling rate, signaling time, and communication time of protein residues of protein alphabets of ‘SARS-CoV-2′ and “COVID-19” are represented in Figure 9B,C, respectively. Again, the signaling rate, signaling time, and communication time of protein residues of two SARS-CoV-2 protein alphabets with antibodies/immunological or vaccine-associated roles were generated and represented in Figure 9D.

Similarly, the 2D maps were generated from the signaling rate, signaling receiving time, and signaling communication time of residue of non-SARS-CoV-2 proteins, which were used to develop the two slogans (Figure S7).

### 3.6. Post-Processing and Decision

**Figure 10:**
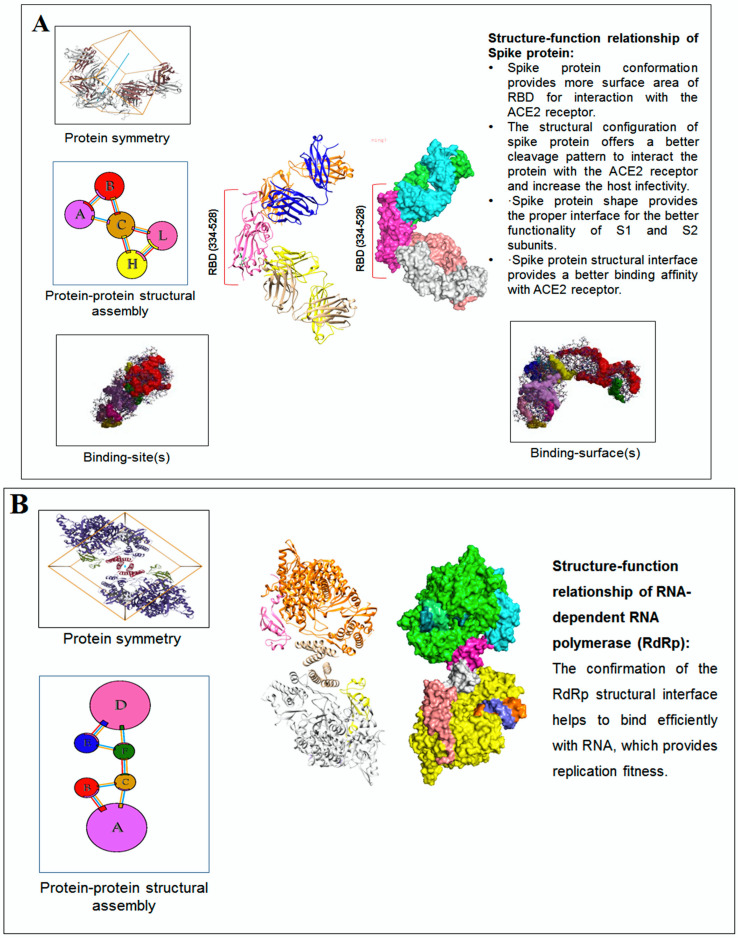

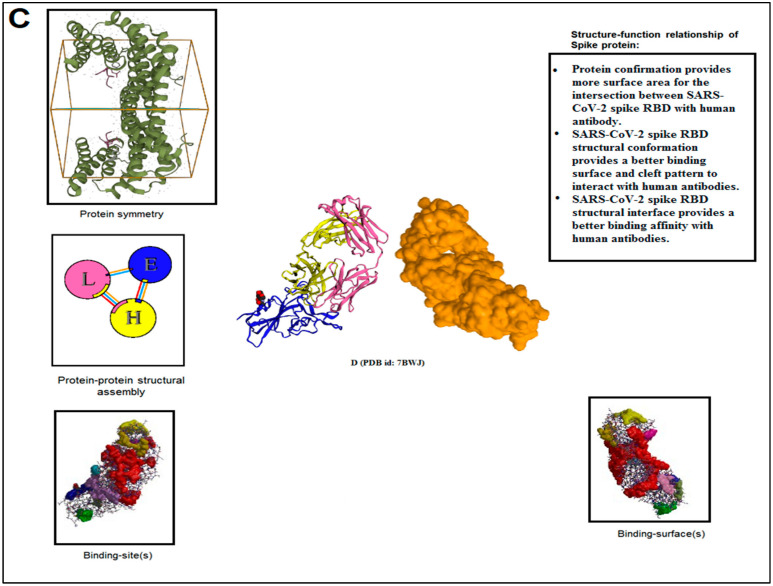
The post-processing and decision-making of the study finally tried to capture the structure–function relationship of the SARS-CoV-2 proteins and non-SARS-CoV-2 proteins. We have represented two examples from the study. (**A**) The figure illustrated the structure–function relationship of the protein alphabet ‘C’ (PDB ID: 6XC3). It is a SARS-CoV-2 S-glycoprotein. (**B**) The figure represented the structure–function relationship of the protein alphabet ‘S’ (PDB ID: 7OYG). It is a SARS-CoV-2 RNA-dependent RNA polymerase (RdRp), which is dimeric form. (**C**) The figure represented the structure–function relationship of the protein alphabet ‘D’ (PDB ID: 7BWJ). The structure represents the human nAb (neutralizing antibodies) linked with SARS-CoV-2 RBD.

The structure–function relationship of SARS-CoV-2 proteins was developed and fine-tuned to their functionality. The protein alphabet ‘C’ (PDB ID: 6XC3) is a complex SARS-CoV-2 S-glycoprotein in the RBD receptor binding domain. The structural conformation of S-glycoprotein provides different functions, such as: (i) It provides more surface area of RBD for interaction with ACE2 receptor. (ii) The structural conformation of spike protein offers a better cleavage pattern and, thus, increases the host infectivity. (iii) The spike protein shape provides proper functional interface for S1 and S2 subunits. (iv) The structural interface provides a more binding interface and provides more binding affinity with the ACE2 receptor (Figure 10A). The second protein alphabet ‘S’ (PDB ID: 7OYG) is a SARS-CoV-2 RNA-dependent RNA polymerase (RdRp) with a dimeric form. The structural conformation of RdRp is responsible for its functionality, and the confirmation of the RdRp structural interface helps to bind efficiently with RNA that provides replication fitness (Figure 10B). The third protein alphabet, ‘D’ (PDB ID: 7BWJ), is a human nAb (neutralizing antibodies) and SARS-CoV-2 RBD interaction structure. The structural conformation of human nAb and SARS-CoV-2 RBD is responsible for its functionality and the confirmation of the antibodies/immunological or vaccine-associated protein structural interface, which helps to bind efficiently with nAb (Figure 10C).

## 4. Discussion

Pattern recognition engages the collection of information based on observations of particular objects consistently [53]. It also tries to collect information from a biological system, such as the symmetrical pattern from the sequence or structure [54,55,56]. Using different category of algorithms, our study explored the protein alphabets fetched from SARS-CoV-2 proteins and non-SARS-CoV-2 proteins to recognize their pattern based on the structural prototype and their functional pattern to create the final structure–function relationship. Twelve SARS-CoV-2 proteins and 14 non-SARS-CoV-2 proteins formed the English alphabet-like structural patterns to design words and slogans.

In a previous study, Howarth searched the PDB and developed the proteins alphabets using 3D structure [57]. To create the words “SARS-CoV-2” and “COVID-19”, our study used only SARS-CoV-2 proteins from PDB and non-SARS-CoV-2 proteins other than Howarth’s protein alphabets to develop the two slogans. The biological functioning of proteins alphabets was used to create the words and catchphrases (Tables S1–S3). Our study concludes that the “C” shaped protein is more complex (PDB ID: 6XC3), and it is a SARS-CoV-2 receptor binding domain with two antibodies, CR3022 and CC12.1. Similarly, the “O” shaped protein is a jointly connected protein of their complexes (PDB ID: 6ZDG). The associated three complexes represented spike ectodomain, which is a bound Fab protein (EY6A Fab).

In recent work, Cicaloni et al. have demonstrated research on cross-reactive T cell recognition between circulating common cold coronaviruses and SARS-CoV-2, including the most recent variants, Delta and Omicron. Further, a deep learning approach based on Siamese networks was used to suggest accurately and efficiently calculate a BLAST-like similarity score between protein sequences. Researchers also tested a neural network model for aligning protein structures. This Siamese long short-term memory model was trained to score the alignments based on BLAST supervision and tested on the set of COVID-19 proteins previously analyzed [58]. However, our study informed similarities of the structural pattern of some SARS-CoV-2 and non-SARS-CoV-2 proteins with the English alphabet. No such potential similarities or specified divergence was found in our study. At the same time, our study has shown the structural relationships between the protein alphabets of SARS-CoV-2 and COVID-19. Finally, our study has shown the structural relationships of SARS-CoV-2 protein alphabets with antibodies/immunological or vaccine-associated roles.

The question however is: does nature favours a biased form of a particular shape of a protein? The protein molecules are often fine-tuned through the evolutionary process, and the particular shape of a protein is often generated through natural selection to provide its proper functionality via folding process [59,60]. The particular alphabet-shaped structures of SARS-CoV-2 proteins and the non-SARS-CoV-2 proteins appear to have been generated for their proper functionality with the evolutionary process. However, deeper analysis may provide details on the structural information and the folding pattern of a protein. Taujale et al. performed an in-depth analysis of glycosyltransferases (GT) families and described the folding design of GT-A. They narrated the complex relationships between regulation structure and function related to GT-A fold for the first time by providing an internal working of the evolutionary framework [61]. Natural selection creates different shapes of proteins, according to their functionality, related to the folding process to ultimately create this type of shape. Based on the local similarity, Hvidsten et al. showed the structure–function relationship of a protein. It has been illustrated that the structure and function relationship of a protein is a significant factor [62]. Our study has provided an extensive understanding of the structure and function relationship of protein. However, the structure and function relationship made a framework with protein evolution that improves the structure sophisticatedly to boost efficient functionality. Additionally, from the analysis of six unique structural families, Taylor and Stoddard found a triangular relationship of three factors: the structural, functional, and evolutionary relationships of a protein [63].

## 5. Limitation of the Study

The considerable challenge of pattern recognition remains in algorithm selection in bioinformatics. The algorithm should help to identify the primary structural pattern linked with the function at features, such as active sites, functional domains, etc. At the same time, proper model building and analysis of protein pattern recognition are essential. Although our study used several models or algorithms, such as deep AI model assembly enumeration, GNM (Gaussian network model), ANM (anisotropic network model), and Markovian stochastic model, for understanding the structural and functional similarity of the protein, detailed analysis of future work is necessary with next-generation algorithms.

## 6. Conclusions

Structural protein pattern alphabets have three important implications. First, the structures draw the concentration of new learners in structural biology studying 3D design of protein and PDB. Secondly, they are excellent examples for the natural creation of protein patterns. Finally, the result of the protein patterns implies the natural selection of these proteins, due to their functional importance. We conclude that, due to the unique function of these proteins, specific structural patterns were developed as a result of natural selection. However, pattern recognition remains a critical area of integrative bioinformatics that can be used to determine structural patterns of SARS-CoV-2 proteins and non-SARS-CoV-2 proteins. It will be a next-generation toolkit for the determination of the structure–function paradigm. This computational approach may assist in solving patterns related to the structural aspect of protein and help to decipher the riddles and puzzles of the complex structure–function relationships of protein and be an important area of modern biology. This area might promise to capture the evolutionary information of proteins and the potential for success in future work.

## 7. Perspectives

(i) **Importance of the field.** Pattern recognition is a rapidly developing field with enormous applicability in biological sciences. This study tried to understand the pattern identification of SARS-CoV-2 proteins. Finally, the study presents new information on the pattern identification of SARS-CoV-2 proteins.
(ii) **A summary of the current thinking.** We have searched for protein-like alphabets involving 3D structure of SARS-CoV-2 from PDB and created two words, “SARS- CoV-2” and “COVID-19”. We have also developed two slogans using non-SARS-CoV-2 proteins, and the slogans are “Vaccinate the world against COVID-19” and “Say no to SARS-CoV-2”. We have used 12 SARS-CoV-2 proteins and 14 non-SARS-CoV-2 proteins to design those words and slogans. We have performed image comparison with protein-like alphabets with English alphabets using the deep AI model. The structural symmetry analysis indicates alphabet-shaped symmetric proteins, such as C, O, I, Hyphen (-), 1(One), S, and A. To determine the dynamics of the structural proteome, we evaluated the inter-residue contact by developing inter-residue contact models with both residue and chain and illustrated the cross-correlations between residues through a cross-correlation (CC) map. In order to understand the residue functionality of proteins, we analyzed the communication/signaling sites of protein residue and signal communication/signal receiving rate of protein alphabets. The assembly enumeration algorithm, anisotropic network model, Gaussian network model, Markovian stochastic model, and other integrative bioinformatics approaches, and tools were used to depict the structural and functional relationships of the protein alphabets of SARS-CoV-2 and COVID-19. After image comparison of protein-like alphabets, the distance score of “I” was the lowest with 22, and “9” was the highest with 40. For post-processing and decision, two protein alphabets were evaluated, protein alphabet “C” (PDB ID: 6XC3) and alphabet “S” (PDB ID: 7OYG), and we understood the structural, functional, and evolutionary relationships using modeling approaches.
(iii) **Future directions.** This study sheds further light on the uniqueness in the functionality of SARS-CoV-2 proteins. The evolutionary process appears to enhance the protein structure smoothly to provide suitable functionality shaped by natural selection. The computational approach may assist in solving patterns related to the structural aspects of other proteins and help to decipher the riddles and puzzles involving the complex structure–function relationships of proteins, which is an important area of modern biology. It has a great promise for capturing the evolutionary information of proteins and the potential for success in future work. It might help to understand the therapeutic target protein pattern, which will be beneficial as a potential therapeutic target discovery.

## Figures and Tables

**Table 1:** The pattern of proteins used in the image comparison study using the protein having antibodies/immunological or vaccine-associated roles.

Sl. No	3D Structure of the Protein Alphabet Compared with English Alphabet	PDB ID	Remarks	Reference
1.	A	7JVC	SARS-CoV-2 spike RBD immunodominant sites in complex with the S2A4 neutralizing antibody Fab fragment	[49]
2.	A	7CWT	Human antibody cocktails (hb27 and fc05 Fab) protein complex with SARS-CoV-2 spike protein	[50]
3.	D	7BWJ	SARS-CoV-2 spike protein (S1 domain) attached with human antibody (heavy and light chain of Ab)	[51]
4.	Y	7R6X	Complex structure of SARS-CoV-2 RBD protein complex with S2E12 Fab, S309 Fab, and S304 Fab domain of Ab	[52]

## Data Availability

All data are available in the main text or the Supplementary Materials.

## Supplementary Materials

The following supporting information can be downloaded at: https://www.mdpi.com/article/10.3390/vaccines11010038/s1. Figure S1: Symmetry structure of non-SARS-CoV-2 proteins which was used to develop two slogans. (A) V-shaped protein alphabet, (B) A-shaped protein alphabet, (C) C-shaped protein alphabet, (D) I-shaped protein alphabet, (E) N-shaped protein alphabet, (F) T-shaped protein alphabet, (G) E-shaped protein alphabet, (H) H-shaped protein alphabet, (I) W-shaped protein alphabet, (J) O-shaped protein alphabet, (K) R-shaped protein alphabet, (L) S-shaped protein alphabet, (M) Y-shaped protein alphabet, (N) L-shaped protein alphabet. Figure S2: Evolutionary protein interface of non-SARS-CoV-2 proteins which was used to develop the two slogans. (A) V-shaped protein alphabet, (B) A-shaped protein alphabet, (C) D-shaped protein alphabet, (D) E-shaped protein alphabet, (E) I-shaped protein alphabet, (F) N-shaped protein alphabet, (G) O-shaped protein alphabet, (H) Y-shaped protein alphabet, (I) E-shaped protein alphabet, (J) W-shaped protein alphabet. Figure S3: Protein-protein interfaces 3D structures of non-SARS-CoV-2 proteins which was used to develop the two slogans. (A) V-shaped protein alphabet, (B) A-shaped protein alphabet, (C) D-shaped protein alphabet, (D) E-shaped protein alphabet, (E) I-shaped protein alphabet, (F) N-shaped protein alphabet, (G) O-shaped protein alphabet, (H) Y-shaped protein alphabet, (I) E-shaped protein alphabet, (J) T-shaped protein alphabet. Figure S4: Inter-residue contact model of all residues and chains of 3D structures of non-SARS-CoV-2 proteins which was used to develop the two slogans. (A) V-shaped protein alphabet, (B) A-shaped protein alphabet, (C) C-shaped protein alphabet, (D) I-shaped protein alphabet, (E) N-shaped protein alphabet, (F) T-shaped protein alphabet, (G) E-shaped protein alphabet, (H) H-shaped protein alphabet, (I) W-shaped protein alphabet. Figure S5: Inter a cross-correlation (CC) map of 3D structures of non-SARS-CoV-2 proteins which was used to develop the two slogans. (A) V-shaped protein alphabet, (B) A-shaped protein alphabet, (C) C-shaped protein alphabet, (D) I-shaped protein alphabet, (E) N-shaped protein alphabet, (F) T-shaped protein alphabet, (G) E-shaped protein alphabet, (H) H-shaped protein alphabet, (I) W-shaped protein alphabet, (J) O-shaped protein alphabet. Figure S6: 2D map for communication/signaling sites and hitting/signal communication times generated with protein residues of 3D structures of non-SARS-CoV-2 proteins which was used to develop the two slogans. (A) V-shaped protein alphabet, (B) C-shaped protein alphabet, (C) N-shaped protein alphabet, (D) T-shaped protein alphabet, (E) H-shaped protein alphabet, (F) W-shaped protein alphabet, (G) L-shaped protein alphabet, (H) S-shaped protein alphabet, (I) Y-shaped protein alphabet. Figure S7: 2D map the signaling rate, signaling receiving time, and signaling communication time from protein residues of 3D structures of non-SARS-CoV-2 proteins which was used to develop the two slogans. (A) V-shaped protein alphabet, (B) C-shaped protein alphabet, (C) N-shaped protein alphabet, (D) T-shaped protein alphabet, (E) E-shaped protein alphabet, (F) H-shaped protein alphabet, (G) W-shaped protein alphabet, (H) L-shaped protein alphabet. Table S1. The various pattern of proteins which were used as the alphabets to develop the word, “SARS CoV-2”. Here, we mentioned the PDB ID and the description of all proteins. Table S2. The various pattern of proteins which were used as the alphabets to develop word, “COVID-19”. Here, we mentioned the PDB ID and the description of all proteins. Table S3. The various pattern of proteins which were used as the alphabets to develop the first slogan, “VACCINATE THE WHOLE WORLD WITH COVID-19 VACCINE.” Here, we mentioned the PDB ID and the description of all proteins. Table S4. The various pattern of proteins which were used as the alphabets to develop the first slogan, “SAY NO TO SARS-CoV-2.” Here, we mentioned the PDB ID and the description of all proteins. Table S5. The generated alphabets and the protein alphabets (Image obtained from PDB) were used in the image comparison study of “SARS CoV-2”. Table S6. The generated alphabets and the protein alphabets (Image obtained from PDB) were used in the image comparison study of “COVID-19”. Table S7. The generated alphabets and the protein alphabets (image obtained from PDB) were used in the image comparison study using the protein having antibodies/immunological or vaccine-associated roles.
