# Supplementary material for: Integrative Bioinformatics Approaches Indicate a Particular Pattern of Some SARS-CoV-2 and Non-SARS-CoV-2 Proteins
Source: Vaccines (Basel). 2022 Dec 23;11(1):38. doi: 10.3390/vaccines11010038 (PMC9864461; doi:10.3390/vaccines11010038)
Supplement: Supplementary file 1 [file vaccines-11-00038-s001.zip › Figure S2.pdf]

Figure S2. Evolutionary protein interface of non-SARS-CoV-2 proteins which was used to develop the two slogans. (A) V-shaped protein alphabet, (B) A-shaped protein alphabet, (C) D-shaped protein alphabet, (D) E-shaped protein alphabet, (E) I-shaped protein alphabet, (F) N-shaped protein alphabet, (G) O-shaped protein alphabet, (H) Y-shaped protein alphabet, (I) E-shaped protein alphabet, (J) W-shaped protein alphabet.

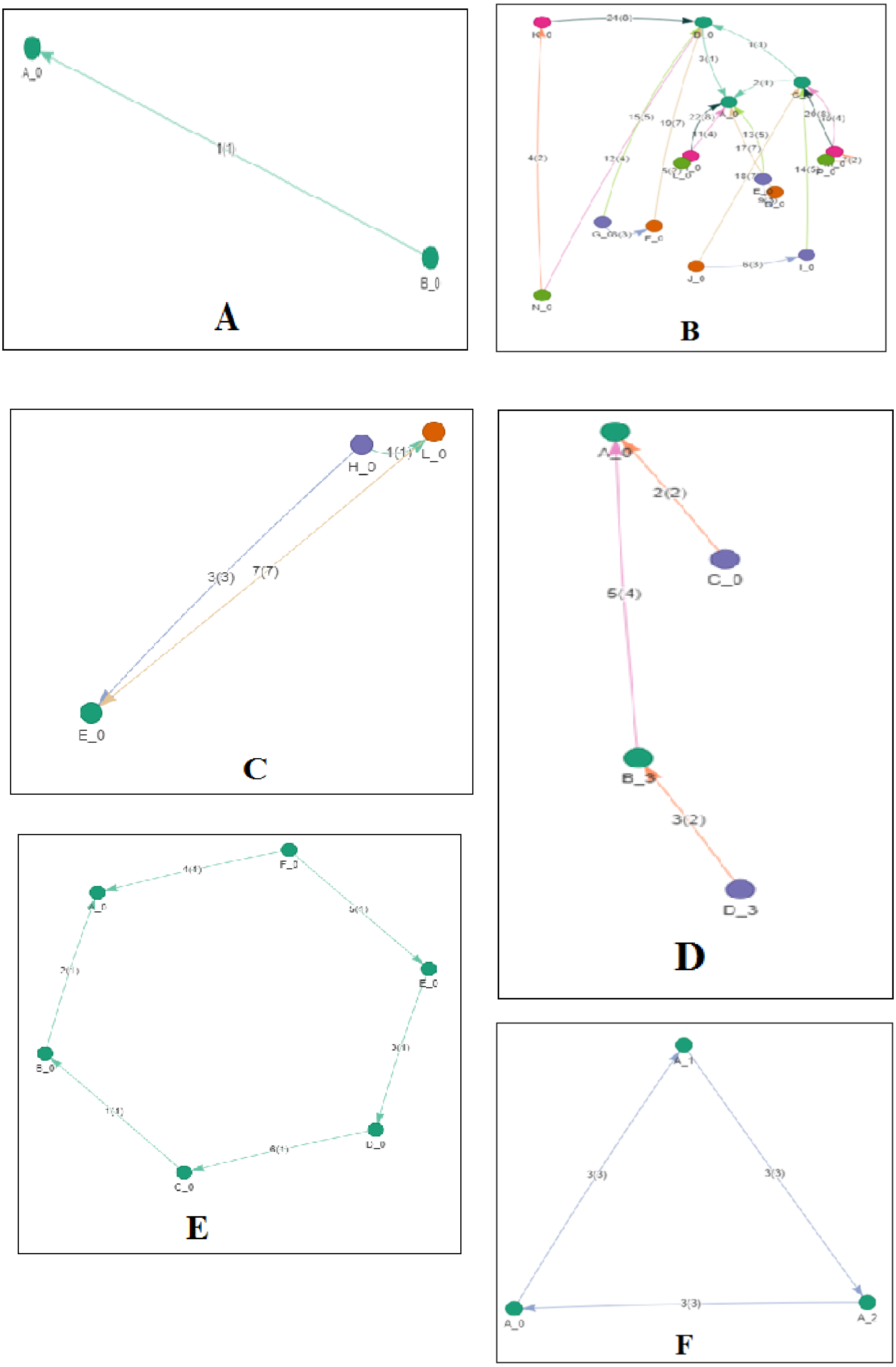

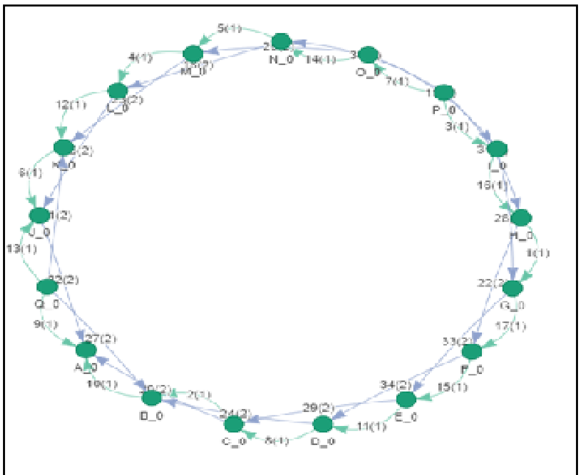

G

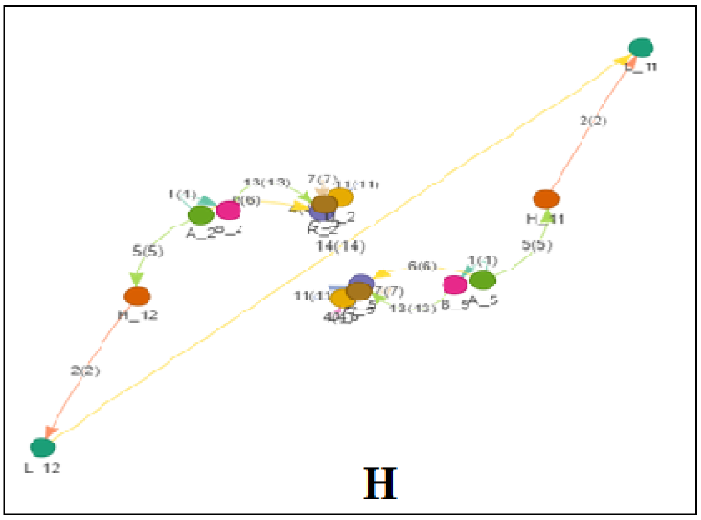

H

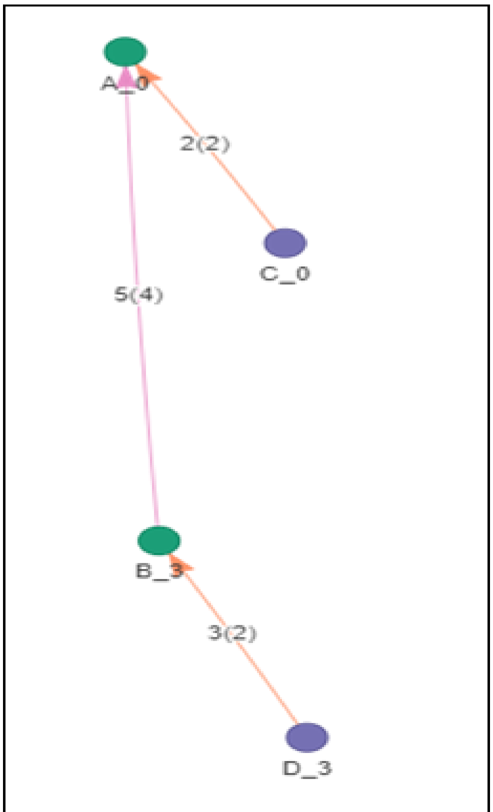

I

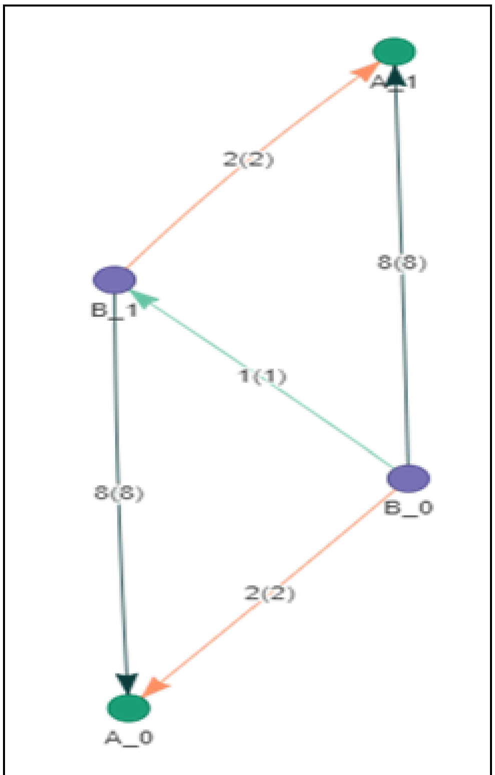

J
