# Supplementary material for: Integrative Bioinformatics Approaches Indicate a Particular Pattern of Some SARS-CoV-2 and Non-SARS-CoV-2 Proteins
Source: Vaccines (Basel). 2022 Dec 23;11(1):38. doi: 10.3390/vaccines11010038 (PMC9864461; doi:10.3390/vaccines11010038)
Supplement: Supplementary file 1 [file vaccines-11-00038-s001.zip › Figure S3.pdf]

Figure S3. Protein-protein interfaces 3D structures of non-SARS-CoV-2 proteins which was used to develop the two slogans. (A) V-shaped protein alphabet, (B) A-shaped protein alphabet, (C) D-shaped protein alphabet, (D) E-shaped protein alphabet, (E) I-shaped protein alphabet, (F) N-shaped protein alphabet, (G) O-shaped protein alphabet, (H) Y-shaped protein alphabet, (I) E-shaped protein alphabet, (J) T-shaped protein alphabet.

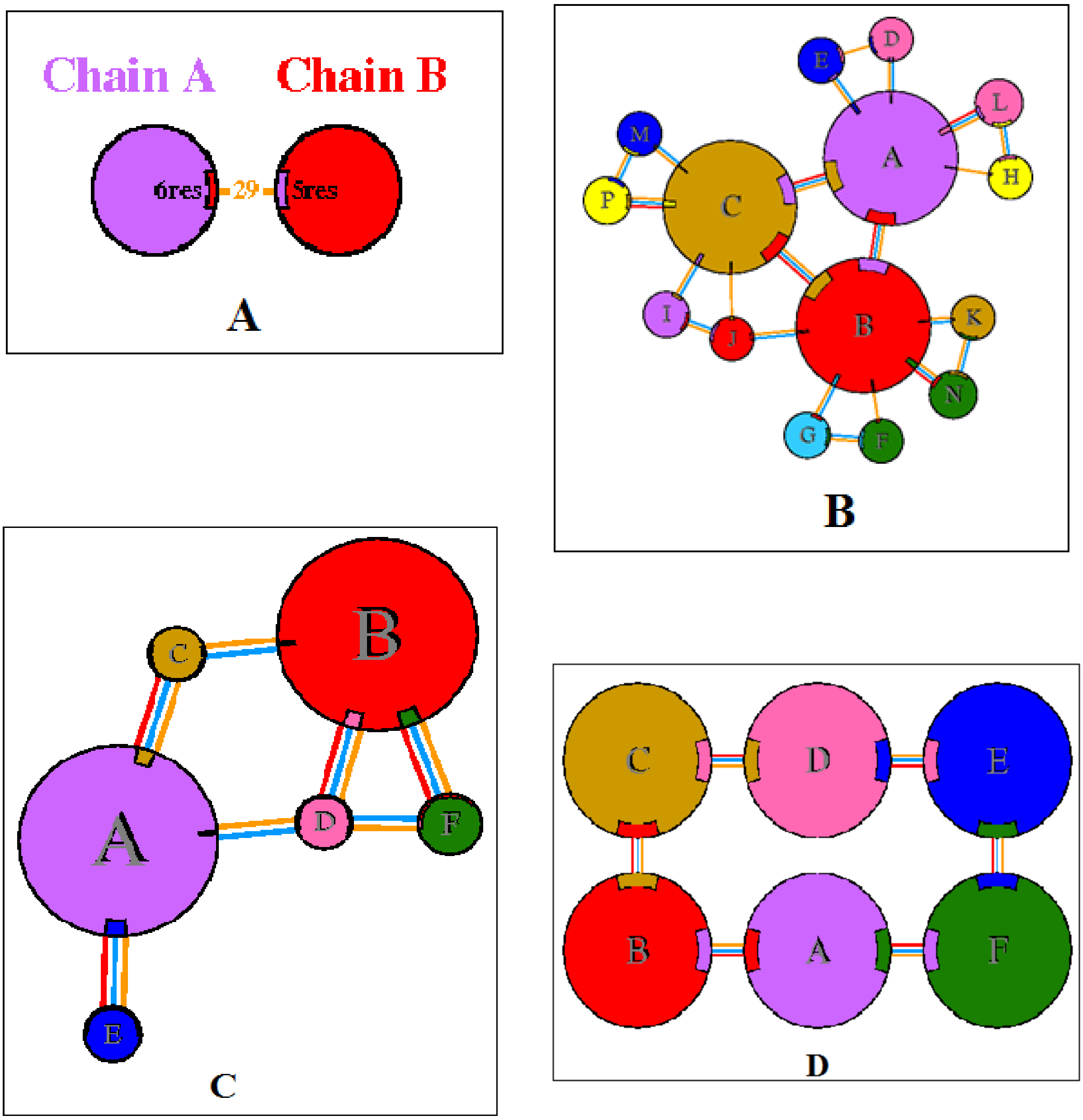

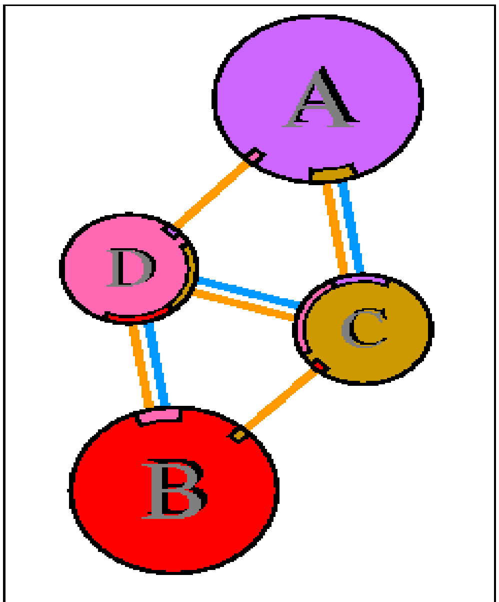
A
D
C
B

E

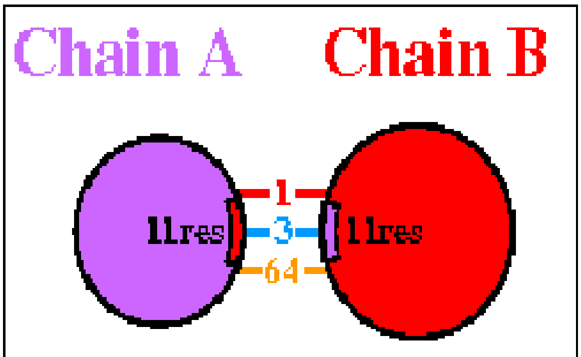
Chain A
Chain B
11res
1
3
64
11res

F

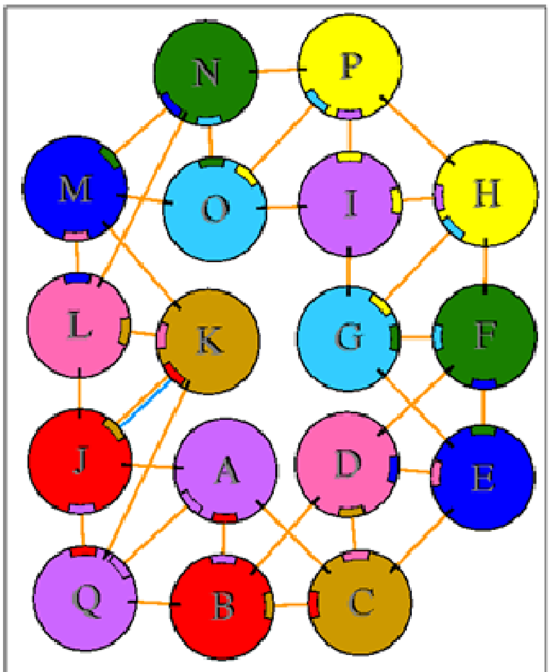
N
P
M
O
I
H
L
K
G
F
J
A
D
E
Q
B
C

G

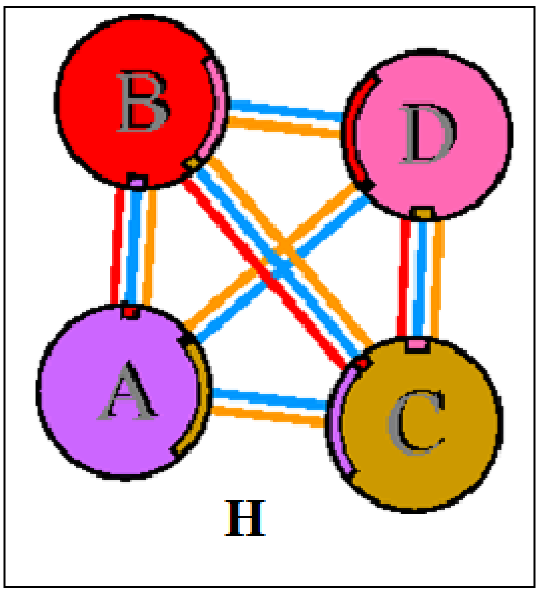
B
D
A
C

H

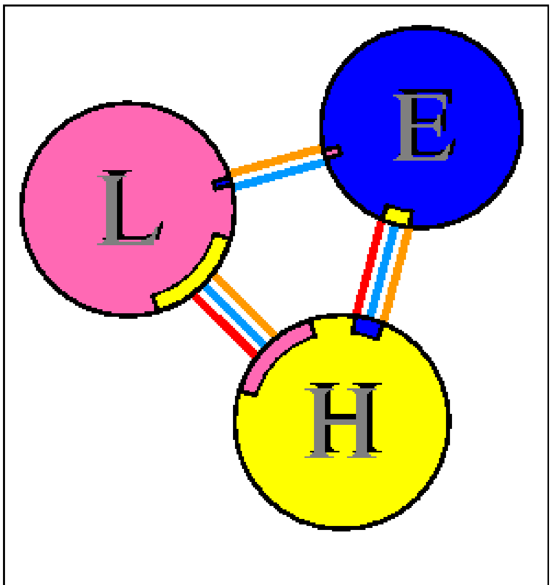
L
E
H

I

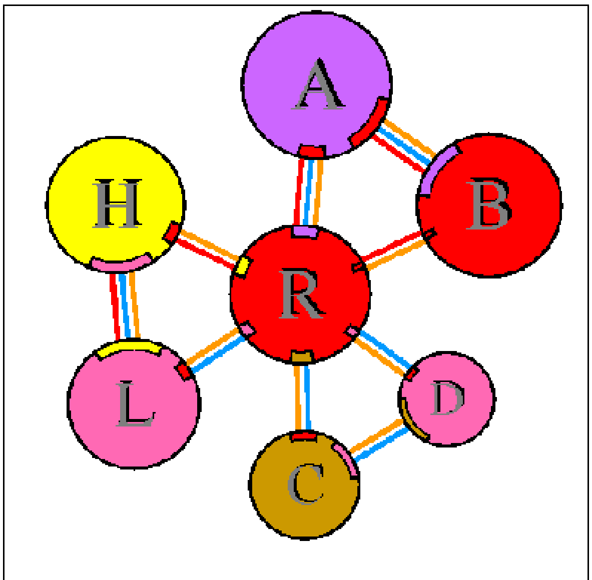
A
H
B
R
L
D
C

J
