# Supplementary material for: Integrative Bioinformatics Approaches Indicate a Particular Pattern of Some SARS-CoV-2 and Non-SARS-CoV-2 Proteins
Source: Vaccines (Basel). 2022 Dec 23;11(1):38. doi: 10.3390/vaccines11010038 (PMC9864461; doi:10.3390/vaccines11010038)
Supplement: Supplementary file 1 [file vaccines-11-00038-s001.zip › Figure S4.pdf]

Figure S4. Inter-residue contact model of all residues and chains of 3D structures of non-SARS-CoV-2 proteins which was used to develop the two slogans. (A) V-shaped protein alphabet, (B) A-shaped protein alphabet, (C) C-shaped protein alphabet, (D) I-shaped protein alphabet, (E) N-shaped protein alphabet, (F) T-shaped protein alphabet, (G) E-shaped protein alphabet, (H) H-shaped protein alphabet, (I) W-shaped protein alphabet

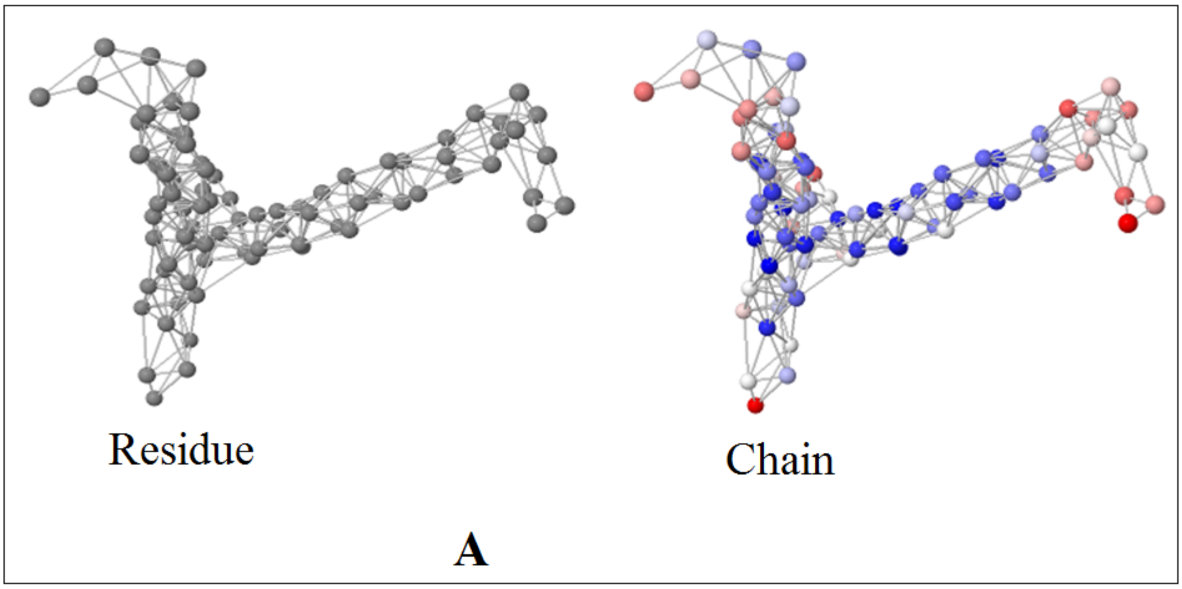

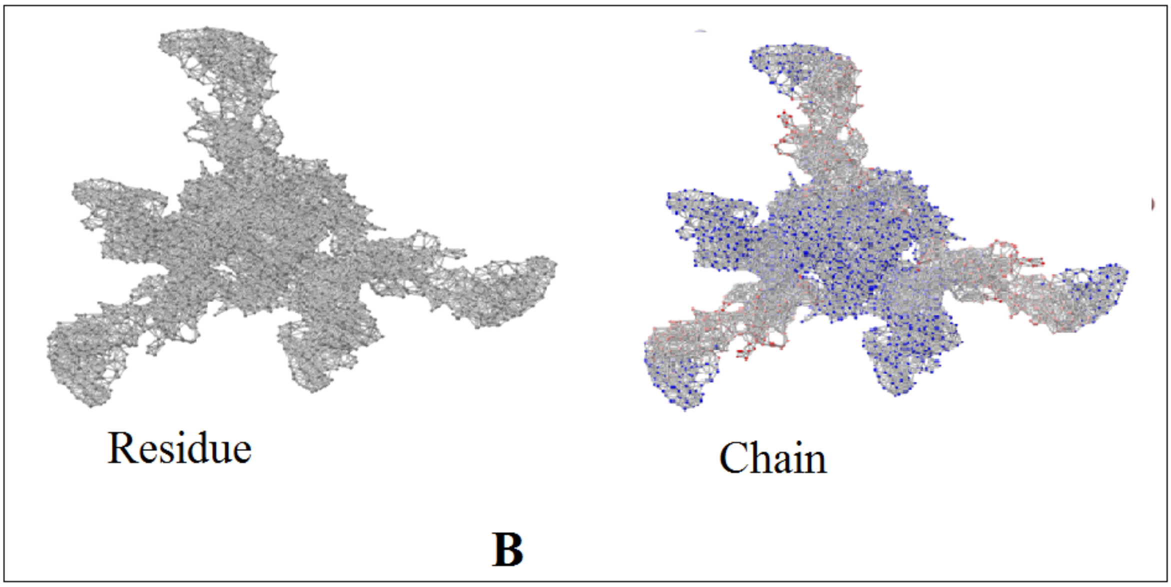

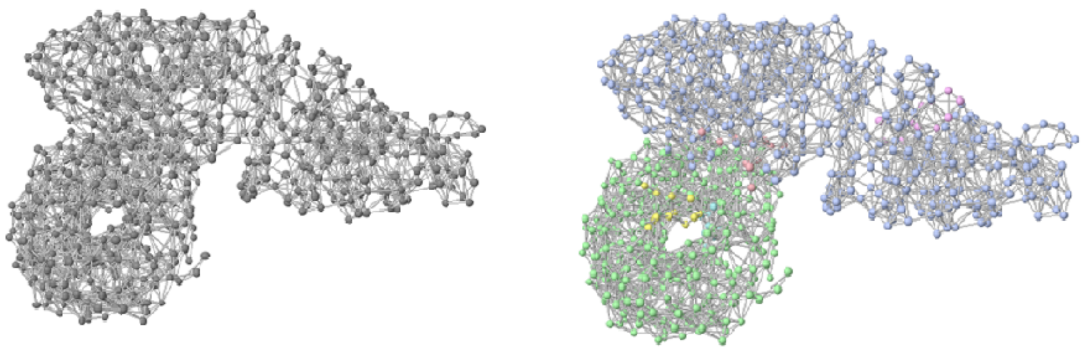

Residue Chain

C

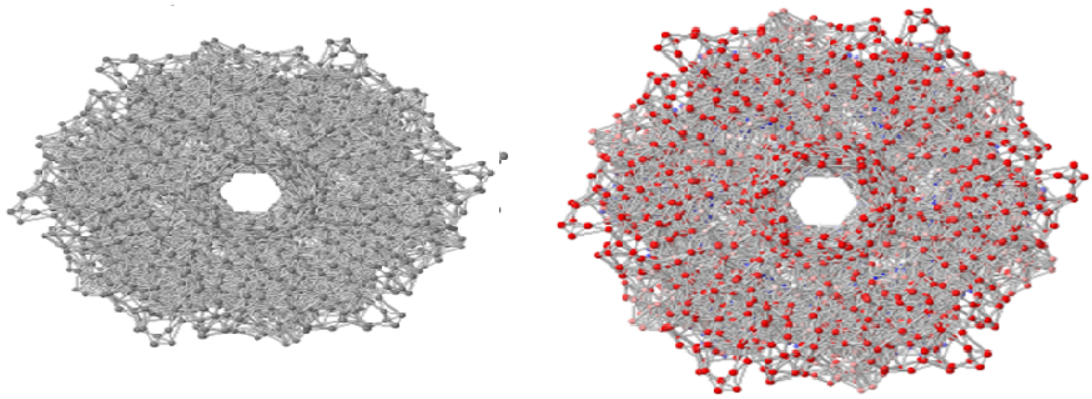

Residue Chain

D

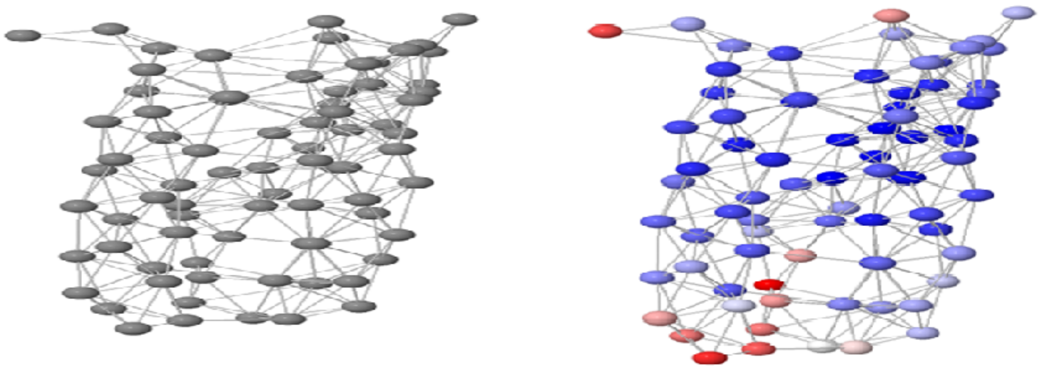

Residue Chain

E

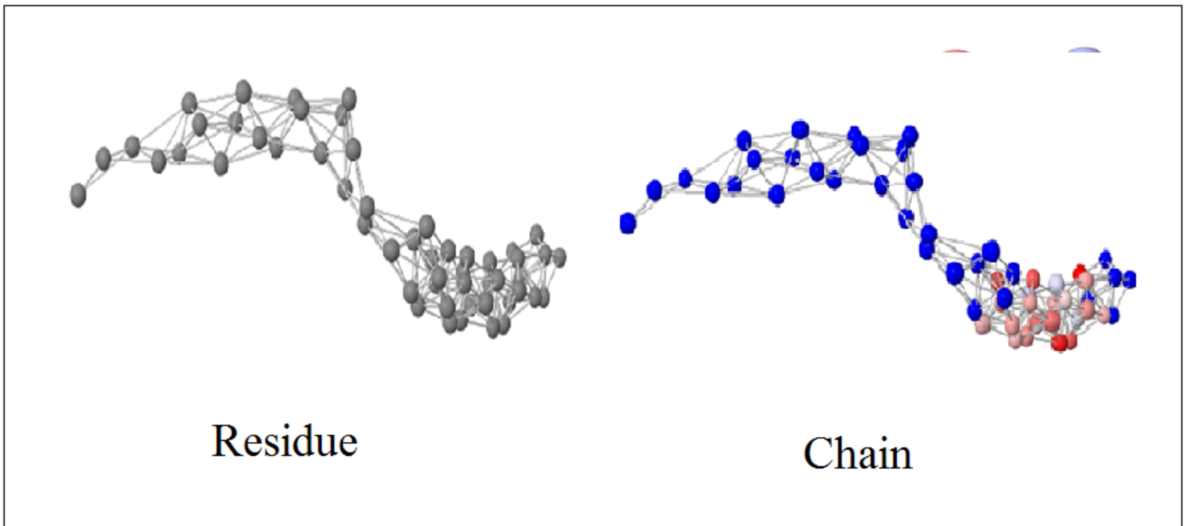

F

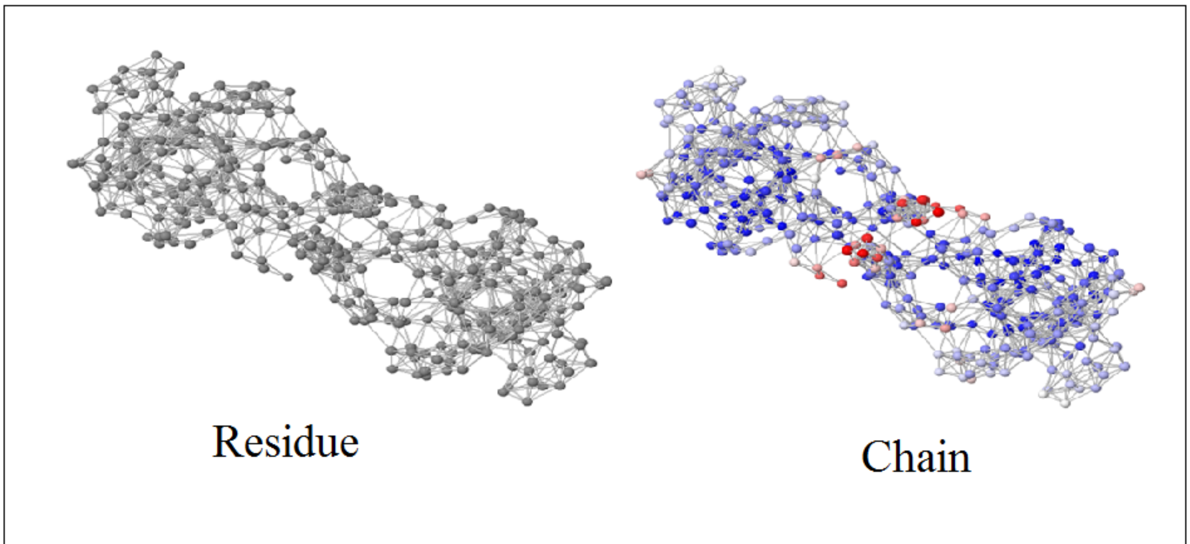

G

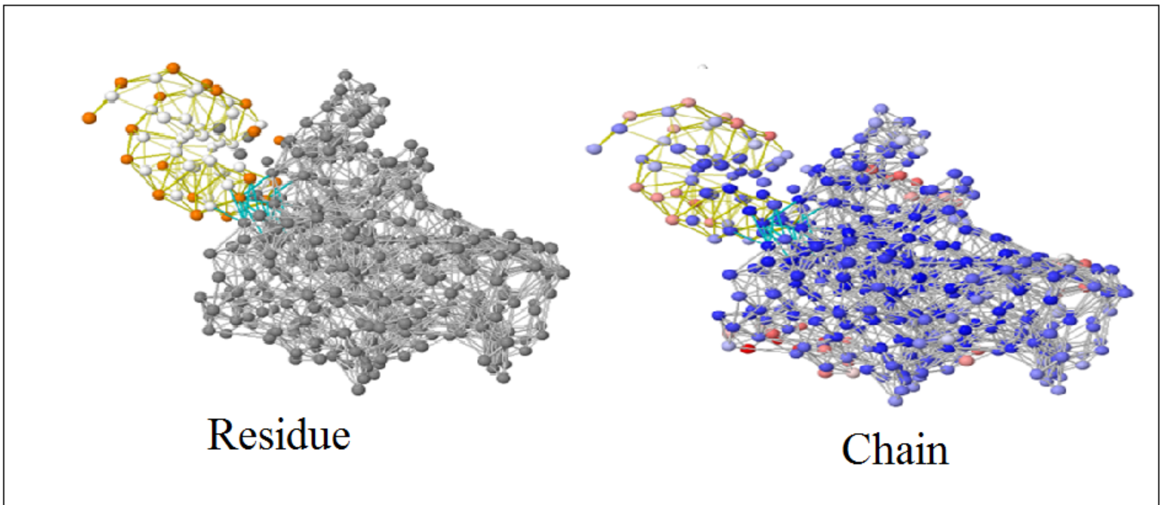

H

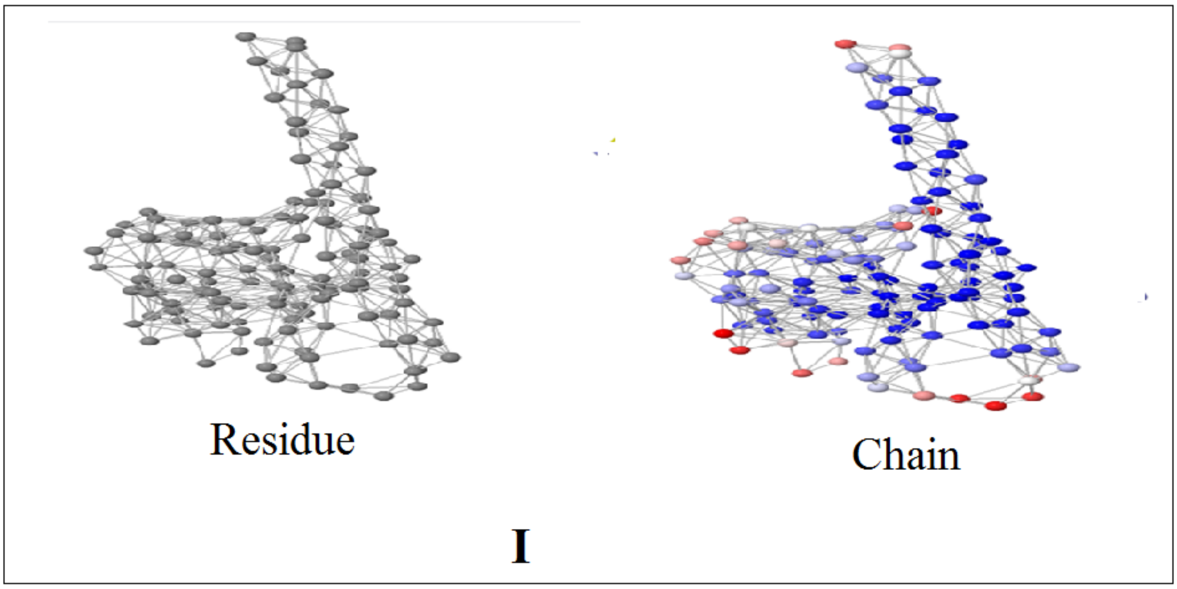
Residue
Chain
I
