# Supplementary material for: Integrative Bioinformatics Approaches Indicate a Particular Pattern of Some SARS-CoV-2 and Non-SARS-CoV-2 Proteins
Source: Vaccines (Basel). 2022 Dec 23;11(1):38. doi: 10.3390/vaccines11010038 (PMC9864461; doi:10.3390/vaccines11010038)
Supplement: Supplementary file 1 [file vaccines-11-00038-s001.zip › Figure S5.pdf]

Figure S5. Inter a cross-correlation (CC) map of 3D structures of non-SARS-CoV-2 proteins which was used to develop the two slogans. (A) V-shaped protein alphabet, (B) A-shaped protein alphabet, (C) C-shaped protein alphabet, (D) I-shaped protein alphabet, (E) N-shaped protein alphabet, (F) T-shaped protein alphabet, (G) E-shaped protein alphabet, (H) H-shaped protein alphabet, (I) W-shaped protein alphabet, (J) O-shaped protein alphabet

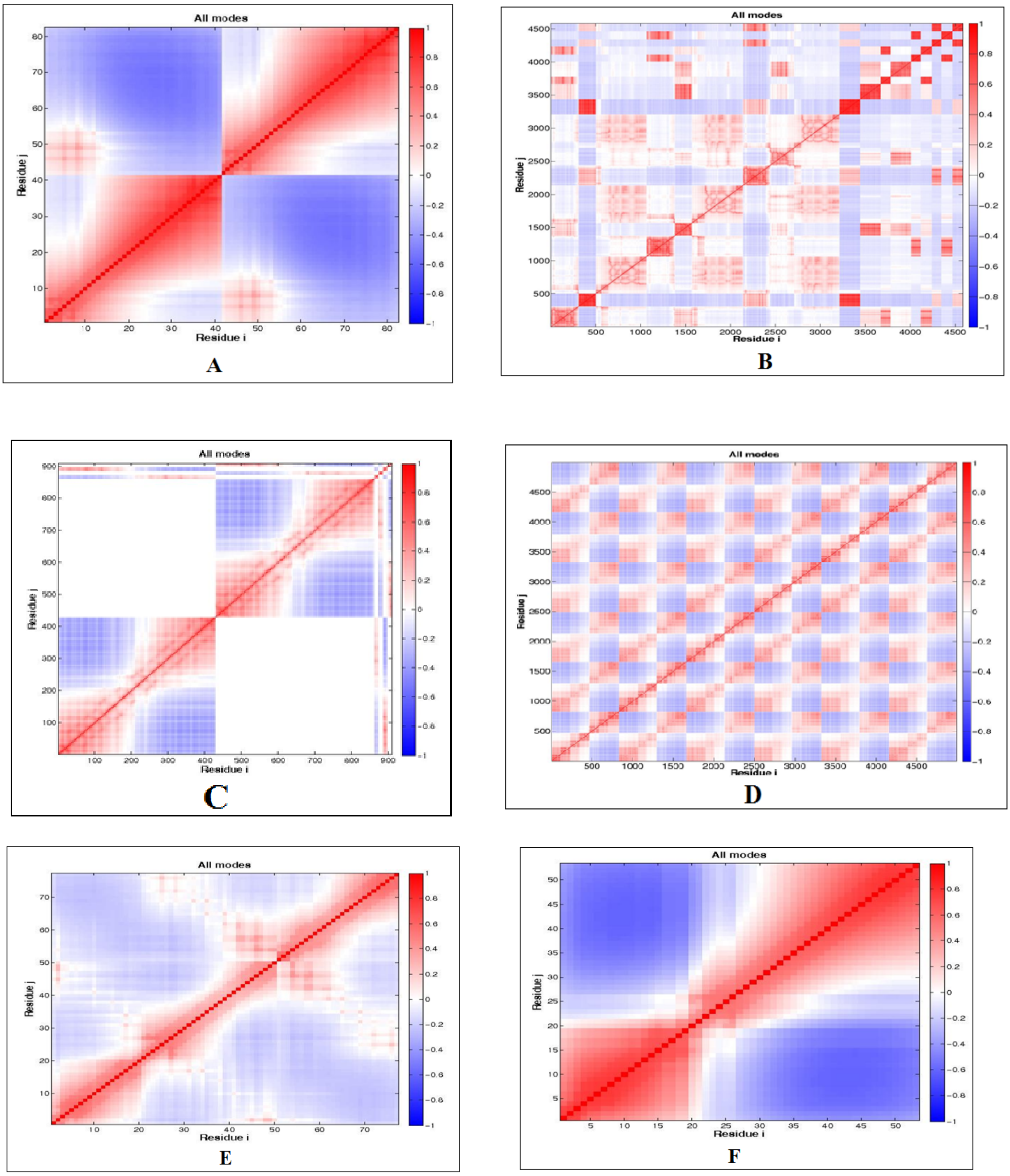

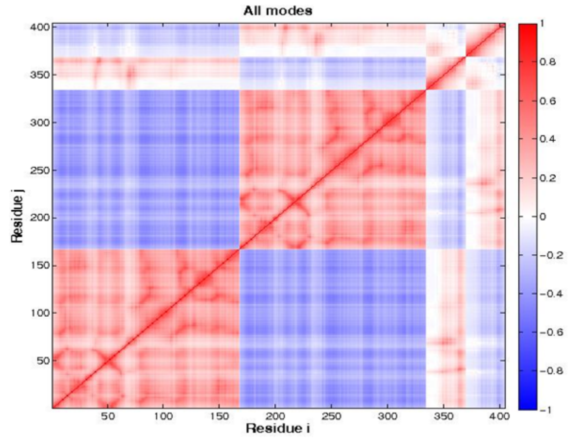
All modes
Residue i
Residue j
50 100 150 200 250 300 350 400
400 350 300 250 200 150 100 50
1 0.8 0.6 0.4 0.2 0 −0.2 −0.4 −0.6 −0.8 −1

G

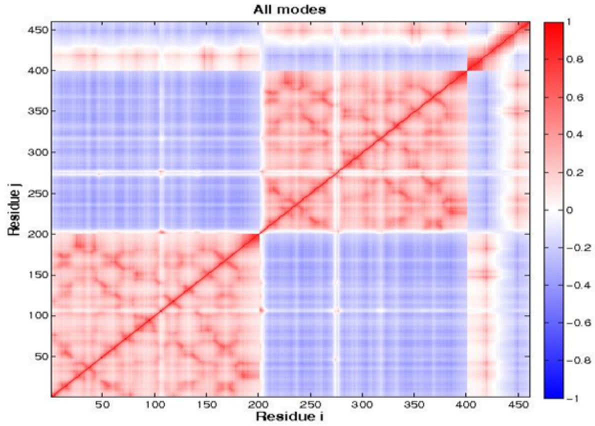
All modes
Residue i
Residue j
50 100 150 200 250 300 350 400 450
450 400 350 300 250 200 150 100 50
1 0.8 0.6 0.4 0.2 0 −0.2 −0.4 −0.6 −0.8 −1

H

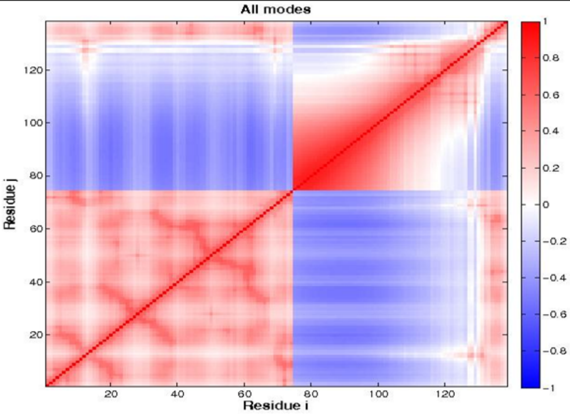
All modes
Residue i
Residue j
20 40 60 80 100 120
120 100 80 60 40 20
1 0.8 0.6 0.4 0.2 0 −0.2 −0.4 −0.6 −0.8 −1

I

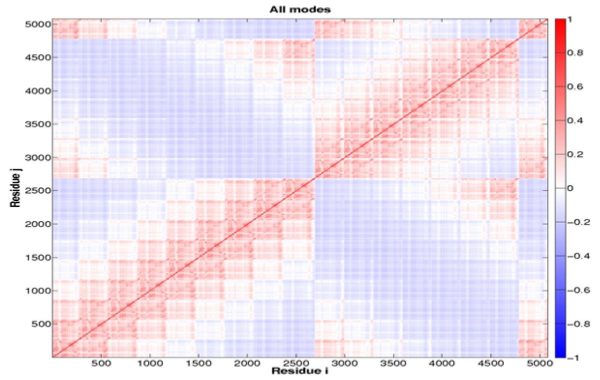
All modes
Residue i
Residue j
500 1000 1500 2000 2500 3000 3500 4000 4500 5000
5000 4500 4000 3500 3000 2500 2000 1500 1000 500
1 0.8 0.6 0.4 0.2 0 −0.2 −0.4 −0.6 −0.8 −1

J
