# Supplementary material for: Integrative Bioinformatics Approaches Indicate a Particular Pattern of Some SARS-CoV-2 and Non-SARS-CoV-2 Proteins
Source: Vaccines (Basel). 2022 Dec 23;11(1):38. doi: 10.3390/vaccines11010038 (PMC9864461; doi:10.3390/vaccines11010038)
Supplement: Supplementary file 1 [file vaccines-11-00038-s001.zip › Figure S6.pdf]

Figure S6. 2D map for communication/signaling sites and hitting/ signal communication times generated with protein residues of 3D structures of non-SARS-CoV-2 proteins which was used to develop the two slogans. (A) V-shaped protein alphabet, (B) C-shaped protein alphabet, (C) N-shaped protein alphabet, (D) T-shaped protein alphabet, (E) H-shaped protein alphabet, (F) W-shaped protein alphabet, (G) L-shaped protein alphabet, (H) S-shaped protein alphabet, (I) Y-shaped protein alphabet.

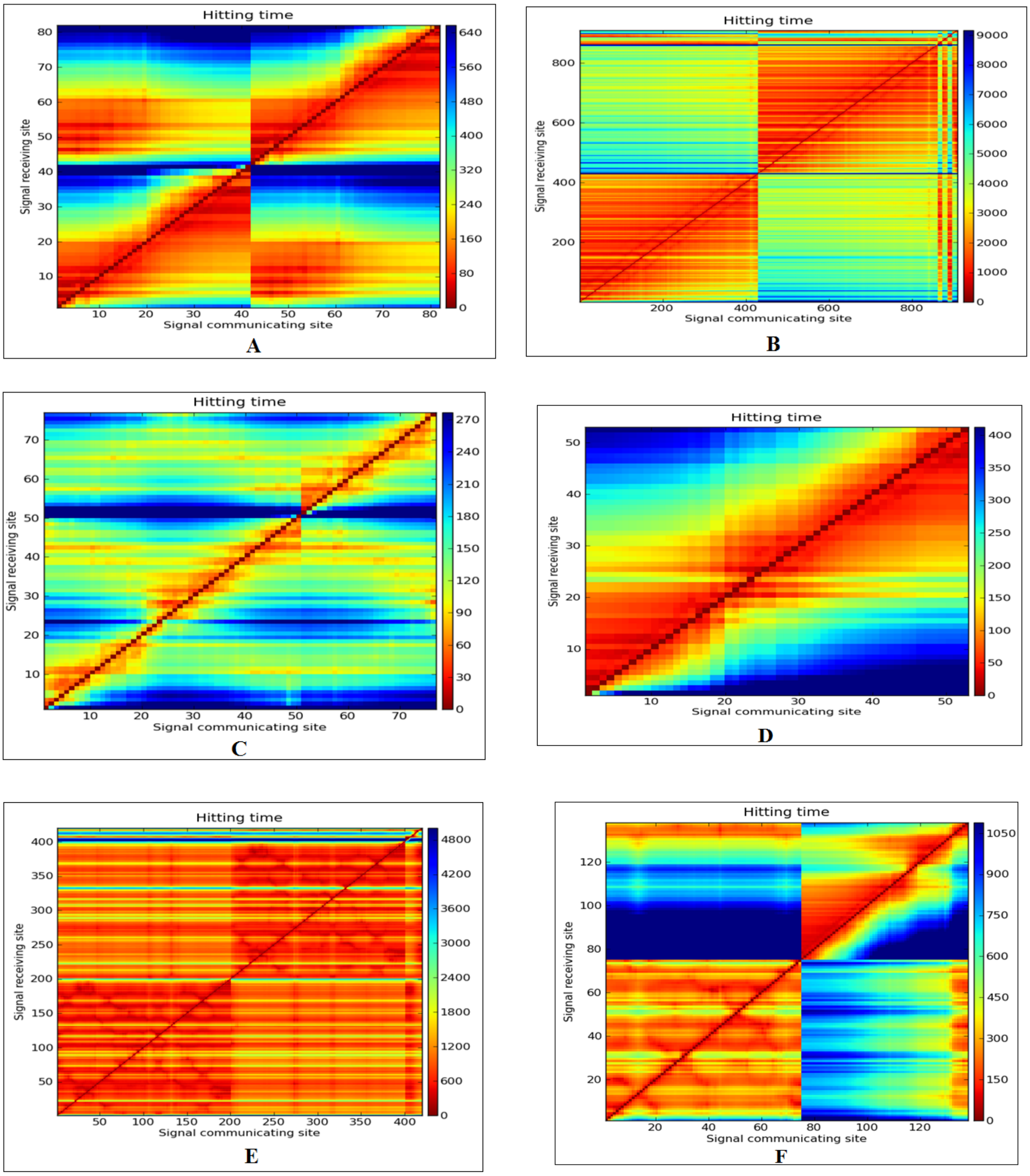

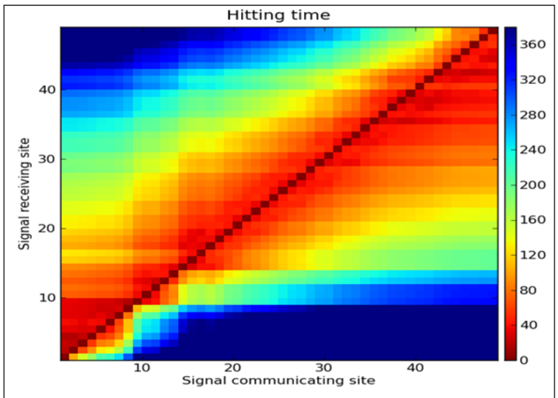
Hitting time
Signal receiving site
Signal communicating site
10
20
30
40
0
40
80
120
160
200
240
280
320
360

G

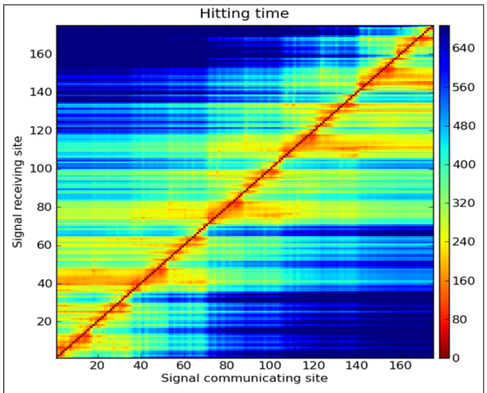
Hitting time
Signal receiving site
Signal communicating site
20
40
60
80
100
120
140
160
0
80
160
240
320
400
480
560
640

H

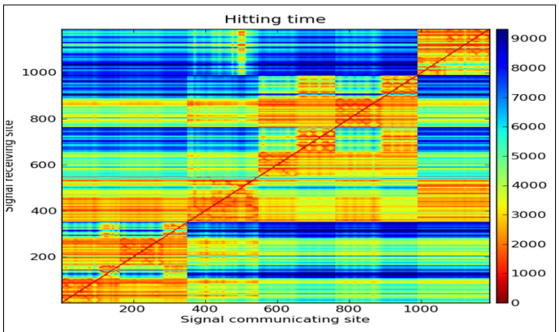
Hitting time
Signal receiving site
Signal communicating site
200
400
600
800
1000
0
1000
2000
3000
4000
5000
6000
7000
8000
9000

I
