# Supplementary material for: Integrative Bioinformatics Approaches Indicate a Particular Pattern of Some SARS-CoV-2 and Non-SARS-CoV-2 Proteins
Source: Vaccines (Basel). 2022 Dec 23;11(1):38. doi: 10.3390/vaccines11010038 (PMC9864461; doi:10.3390/vaccines11010038)
Supplement: Supplementary file 1 [file vaccines-11-00038-s001.zip › Figure S7.pdf]

**Figure S7**. 2D map the signaling rate, signaling receiving time, and signaling communication time from protein residues of 3D structures of non-SARS-CoV-2 proteins which was used to develop the two slogans. (A) V-shaped protein alphabet, (B) C-shaped protein alphabet, (C) N-shaped protein alphabet, (D) T-shaped protein alphabet, (E) E-shaped protein alphabet, (F) H-shaped protein alphabet, (G) W-shaped protein alphabet, (H) L-shaped protein alphabet.

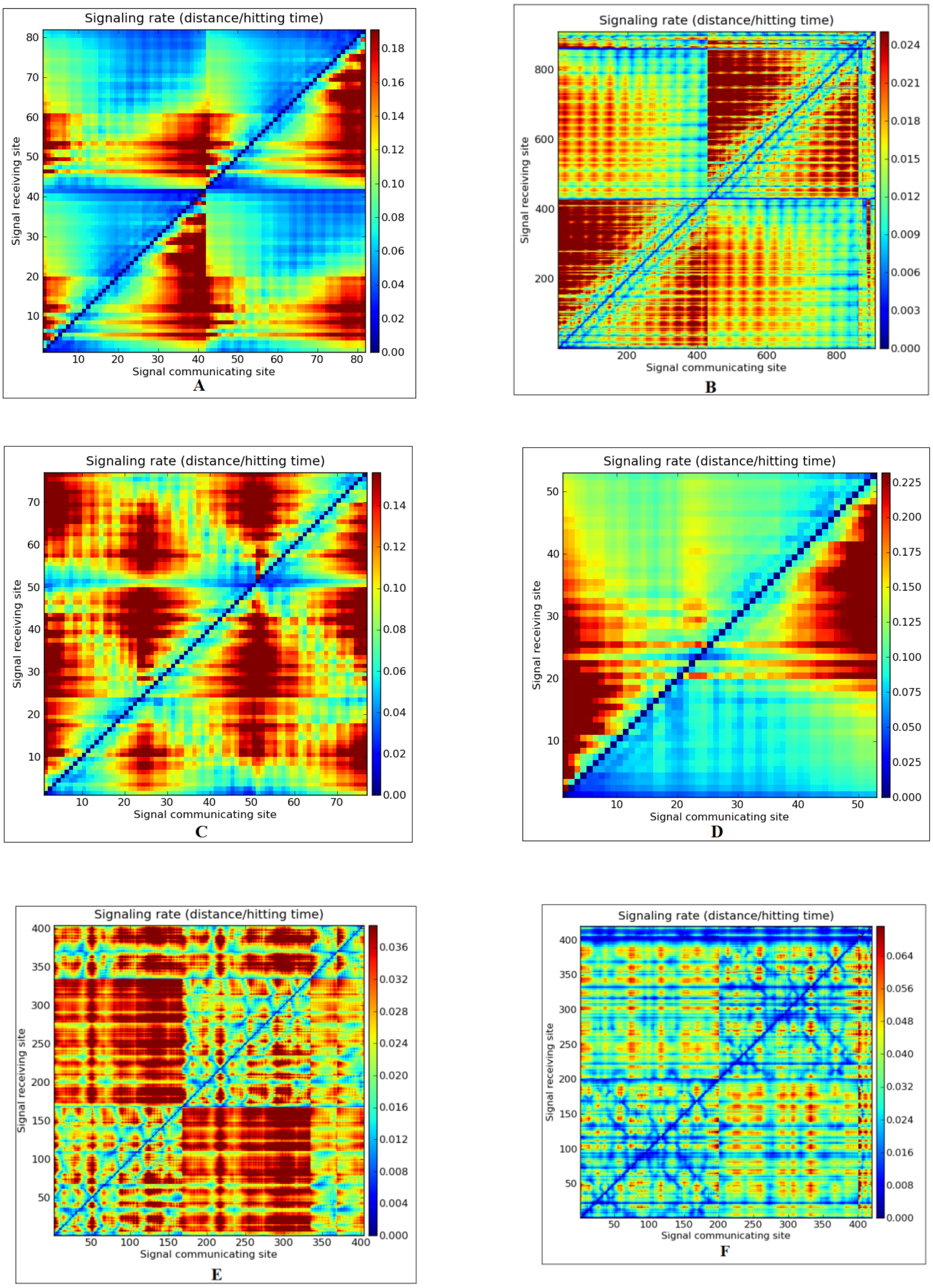

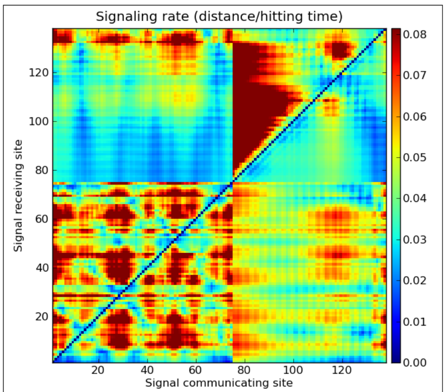

Signaling rate (distance/hitting time)
Signal receiving site
Signal communicating site
20
40
60
80
100
120
0.00
0.01
0.02
0.03
0.04
0.05
0.06
0.07
0.08


**G**

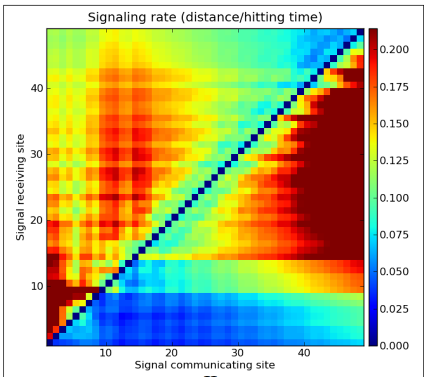

Signaling rate (distance/hitting time)
Signal receiving site
Signal communicating site
10
20
30
40
0.000
0.025
0.050
0.075
0.100
0.125
0.150
0.175
0.200


**H**
