# Supplementary material for: Integrative Bioinformatics Approaches Indicate a Particular Pattern of Some SARS-CoV-2 and Non-SARS-CoV-2 Proteins
Source: Vaccines (Basel). 2022 Dec 23;11(1):38. doi: 10.3390/vaccines11010038 (PMC9864461; doi:10.3390/vaccines11010038)
Supplement: Supplementary file 1 [file vaccines-11-00038-s001.zip › Table S1.pdf]

Table S1. The various pattern of proteins which were used as the alphabets to develop the word, "SARS CoV-2". Here, we mentioned the PDB ID and the description of all proteins.

Sl. No.	Pattern of the 3D structure of the protein which Alphabet/Numbers/ Characters	PDB ID	Image	Description of the protein
1.	S	7OYG	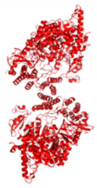	RNA-dependent RNA polymerase protein of SARS-CoV-2 in dimeric form
2.	A	7JVC	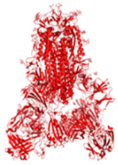	Immunodominant sites containing receptor binding domain protein of SARS-CoV-2
3.	R	7CWL	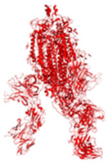	Close conformation of SARS-CoV-2 S-glycoprotein
4.	S	7OYG	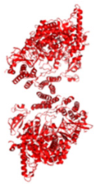	RNA-dependent RNA polymerase protein of SARS-CoV-2 in dimeric form
5.	-	6XRA	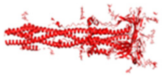	Spike protein of SARS-CoV-2 in distinct conformation
6.	C	6XC3	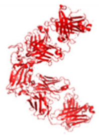	Receptor binding domain complex of SARS-CoV-2 S-glycoprotein
7.	O	6ZDG	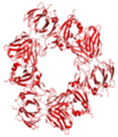	Triple complex of disordered of SARS-CoV-2 spike ectodomain
8.	V	7L7E	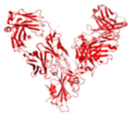	Receptor binding domain protein of SARS-CoV-2 S-glycoprotein

9.	-	6XRA	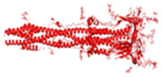	Spike protein of SARS-CoV-2 in distinct conformation
10.	2	7C8D	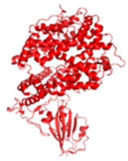	Receptor binding domain protein of SARS-CoV-2 conjugated with cat ACE2 receptor
