# Supplementary material for: Integrative Bioinformatics Approaches Indicate a Particular Pattern of Some SARS-CoV-2 and Non-SARS-CoV-2 Proteins
Source: Vaccines (Basel). 2022 Dec 23;11(1):38. doi: 10.3390/vaccines11010038 (PMC9864461; doi:10.3390/vaccines11010038)
Supplement: Supplementary file 1 [file vaccines-11-00038-s001.zip › Table S2.pdf]

Table S2. The various pattern of proteins which were used as the alphabets to develop word, "COVID-19". Here, we mentioned the PDB ID and the description of all proteins.

Sl. No.	Pattern of the 3D structure of the protein which Alphabet/Numbers/ Characters	PDB ID	Image	Description of the protein
1.	C	6XC3	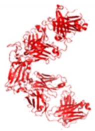	Receptor binding domain complex of SARS-CoV-2 S-glycoprotein
2.	O	6ZDG	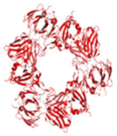	Triple complex of disordered of SARS-CoV-2 spike ectodomain
3.	V	7L7E	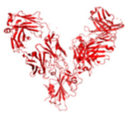	Receptor binding domain protein of SARS-CoV-2 S-glycoprotein
4.	I	6LXT	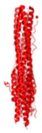	Post fusion core protein of SARS-CoV-2 S2 subunit
5.	D	7BWJ	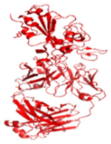	Hetero trimeric protein complex SARS-CoV-2 S-glycoprotein
6.	-	6XRA	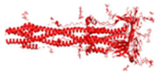	Spike protein of SARS-CoV-2 in distinct conformation
7.	1	7EK6	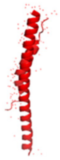	Hetero 6-meric peptides of SARS-CoV-2 virus
8.	9	6YZ7	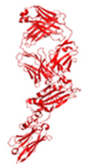	Tetrameric protein of SARS-CoV-2 S-glycoprotein
