# Supplementary material for: Integrative Bioinformatics Approaches Indicate a Particular Pattern of Some SARS-CoV-2 and Non-SARS-CoV-2 Proteins
Source: Vaccines (Basel). 2022 Dec 23;11(1):38. doi: 10.3390/vaccines11010038 (PMC9864461; doi:10.3390/vaccines11010038)
Supplement: Supplementary file 1 [file vaccines-11-00038-s001.zip › Table S3.pdf]

Table S3. The various pattern of proteins which were used as the alphabets to develop the first slogan, "VACCINATE THE WHOLE WORLD WITH COVID-19 VACCINE." Here, we mentioned the PDB ID and the description of all proteins.

Sl. No.	Pattern of the 3D structure of the protein which Alphabet/Numbers /Characters	PDB ID	Image	Description of the protein
1.	V	2K6S	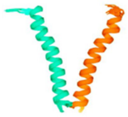	Coiled-coil domain structure of Rab11 interacting proteins in C-terminal region
2.	A	7CWT	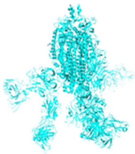	Human antibody cocktails protein complex with SARS-CoV-2 Spike protein
3.	C	5B56	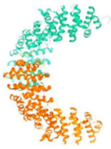	Viral protein R of HIV-type I complexed with DIBB-M-Importin-Alpha subunit 2
4.	C		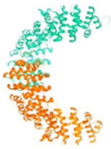	
5.	I	6V0D	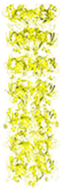	Bacterial envelope-spanning tunnel B type protein
6.	N	6H48	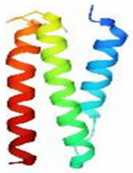	Polyamorous repressor protein in monomeric form of bacteria
7.	A	7CWT	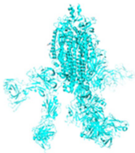	Human antibody cocktails protein complex with SARS-CoV-2 Spike protein

8.	T	2KB7	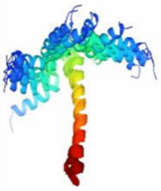	Monomeric structure of bacterial phospholamban protein lipid bilayers
9.	E	2GZD	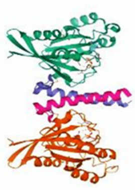	Rab11 family of interacting protein type 2 in human
10.	T	2KB7	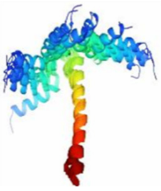	Monomeric structure of bacterial phospholamban protein lipid bilayers
11.	H	5MCT	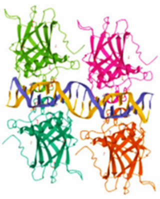	Synthetic construct of homo 4-mer p53 proteins with DNA
12.	E	2GZD	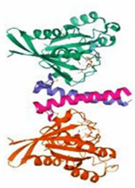	Rab11 family of interacting protein type 2 in human
13.	W	1TXQ	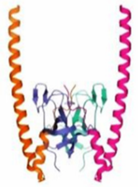	Terminal dimerization domain of EB1 and microtubule binding domain of the dynactin subunit (p150Glued)
14.	H	5MCT	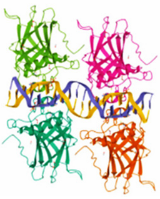	Synthetic construct of homo 4-mer p53 Proteins with DNA
15.	O	6ODJ	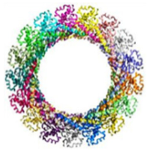	Cyclic periplasmic ring complex of bacterial outer membrane core complex protein

16.	L	2MV6	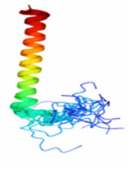	Transmembrane and juxta-membrane domain of erythropoietin receptor protein in dimerization form
17.	E	2GZD	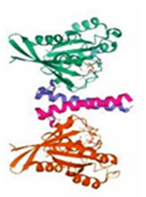	Rab11 family of interacting protein type 2 in human
18.	W	1TXQ	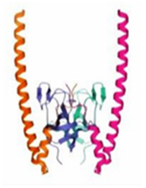	Terminal dimerization domain of EB1 and microtubule binding domain of the dynactin subunit (p150Glued)
19.	O	6ODJ	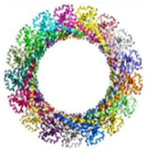	Cyclic periplasmic ring complex of bacterial outer membrane core complex protein
20.	R	2GBK	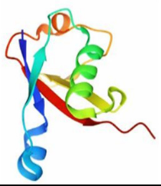	Insertion multinational (9-10 MoaD) structure of Ubiquitin protein
21.	L	2MV6	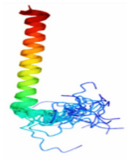	Transmembrane and juxta-membrane domain of erythropoietin receptor protein in dimerization form
22.	D	7BWJ	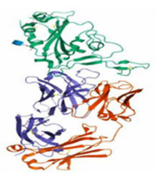	Viral receptor binding domain attached with human antibody
23.	W	1TXQ	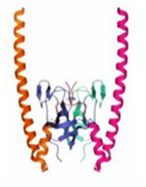	Terminal dimerization domain of EB1 and microtubule binding domain of the dynactin subunit (p150Glued)

24.	I	6V0D	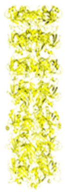	Bacterial envelope-spanning tunnel B type protein
25.	T	2KB7	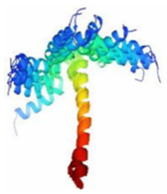	Monomeric structure of bacterial phospholamban protein lipid bilayers
26.	H	5MCT	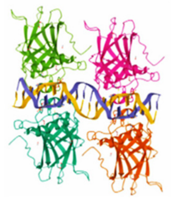	Synthetic construct of homo 4-mer p53 Proteins with DNA
27.	C	6XC3	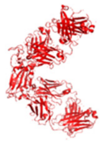	Receptor binding domain complex of SARS-CoV-2 S-glycoprotein
28.	O	6ZDG	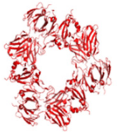	Triple complex of disordered of SARS-CoV-2 spike ectodomain
29.	V	7L7E	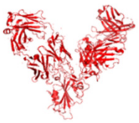	Receptor binding domain protein of SARS-CoV-2 S-glycoprotein
30.	I	6LXT	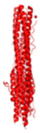	Post fusion core protein of SARS-CoV-2 S2 subunit
31.	D	7BWJ	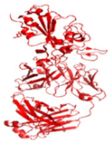	Hetero trimeric protein complex SARS-CoV-2 S-glycoprotein
32.	-	6XRA	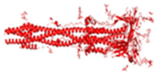	Spike protein of SARS-CoV-2 in distinct conformation

33.	1	7EK6	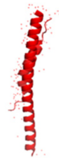	Hetero 6-meric peptides of SARS-CoV-2 virus
34.	9	6YZ7	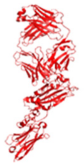	Tetrameric protein of SARS-CoV-2 S-glycoprotein
35.	V	2K6S	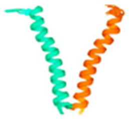	Coiled-coil domain structure of Rab11 interacting proteins in C-terminal region
36.	C	5B56	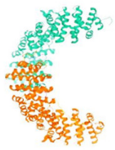	Viral protein R of HIV-type I complexed with DIBB-M-Importin-Alpha subunit 2
37.	C			
38.	I	6V0D	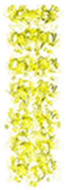	Bacterial envelope-spanning tunnel B type protein
39.	N	6H48	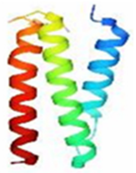	Polyamorous repressor protein in monomeric form of bacteria
40.	E	2GZD	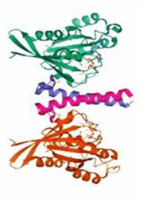	Rab11 family of interacting protein type 2 in human
