# Supplementary material for: Integrative Bioinformatics Approaches Indicate a Particular Pattern of Some SARS-CoV-2 and Non-SARS-CoV-2 Proteins
Source: Vaccines (Basel). 2022 Dec 23;11(1):38. doi: 10.3390/vaccines11010038 (PMC9864461; doi:10.3390/vaccines11010038)
Supplement: Supplementary file 1 [file vaccines-11-00038-s001.zip › Table S4.pdf]

Table S4. The various pattern of proteins which were used as the alphabets to develop the first slogan, "SAY NO TO SARS-CoV-2." Here, we mentioned the PDB ID and the description of all proteins.

Sl. No.	Pattern of the 3D structure of the protein which Alphabet/Numbers/Characters	PDB ID	Image	Description of the protein
1.	S	4OZS	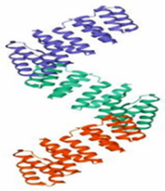	Pentatricopeptide repeat bacterial RNA binding protein
2.	A	7CWT	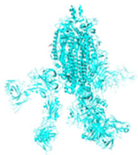	Human antibody cocktails protein complex with SARS-CoV-2 Spike protein
3.	Y	7R6X	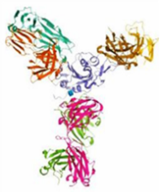	Complex structure of SARS-CoV-2 receptor binding domain protein with potent receptor binding motif antibody
4.	N	6H48	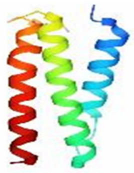	Polyamorous repressor protein in monomeric form of bacteria
5.	O	6ODJ	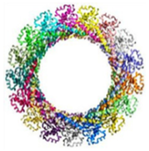	Cyclic periplasmic ring complex of bacterial outer membrane core complex protein
6.	T	2KB7	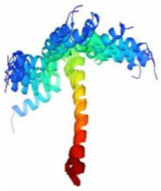	Monomeric structure of bacterial phospholamban protein lipid bilayers
7.	O	6ODJ	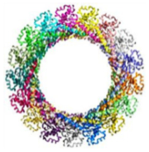	Cyclic periplasmic ring complex of bacterial outer membrane core

				complex protein
8.	S	7OYG	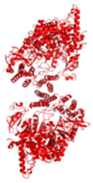	RNA-dependent RNA polymerase protein of SARS-CoV-2 in dimeric form
9.	A	7JVC	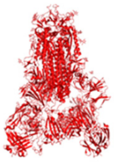	Immunodominant sites containing receptor binding domain protein of SARS-CoV-2
10.	R	7CWL	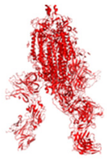	Close conformation of SARS-CoV-2 S-glycoprotein
11.	S	7OYG	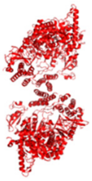	RNA-dependent RNA polymerase protein of SARS-CoV-2 in dimeric form
12.	-	6XRA	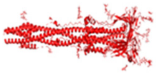	Spike protein of SARS-CoV-2 in distinct conformation
13.	C	6XC3	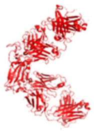	Receptor binding domain complex of SARS-CoV-2 S-glycoprotein
14.	O	6ZDG	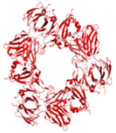	Triple complex of disordered of SARS-CoV-2 spike ectodomain
15.	V	7L7E	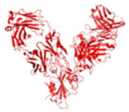	Receptor binding domain protein of SARS-CoV-2 S-glycoprotein
16.	-	6XRA	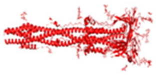	Spike protein of SARS-CoV-2 in distinct conformation

17.	2	7C8D	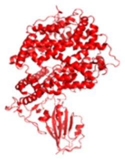	Receptor binding domain protein of SARS-CoV-2 conjugated with cat ACE2 receptor
