# Supplementary material for: Integrative Bioinformatics Approaches Indicate a Particular Pattern of Some SARS-CoV-2 and Non-SARS-CoV-2 Proteins
Source: Vaccines (Basel). 2022 Dec 23;11(1):38. doi: 10.3390/vaccines11010038 (PMC9864461; doi:10.3390/vaccines11010038)
Supplement: Supplementary file 1 [file vaccines-11-00038-s001.zip › Table S5.pdf]

Table S5. The generated alphabets and the protein alphabets (Image obtained from PDB) were used in the image comparison study of "SARS CoV-2".

Sl. No.	Pattern of the 3D structure of the protein as Alphabet/Numbers/ Characters used in this study	Created image of alphabets	Protein alphabets (Image obtained from PDB)
1.	S	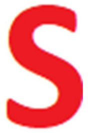	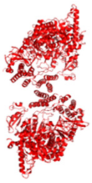
2.	A	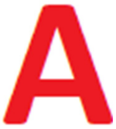	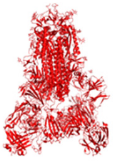
3.	R	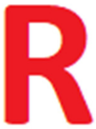	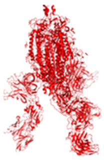
4.	S	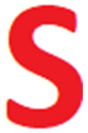	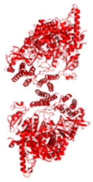
5.	- (Hyphen)	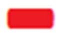	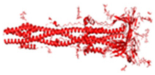
6.	C	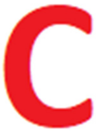	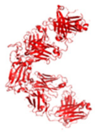
7.	O	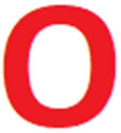	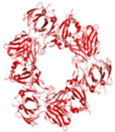
8.	V	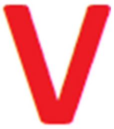	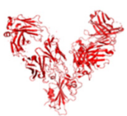

9.	- (Hyphen)	-	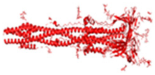
10.	2	2	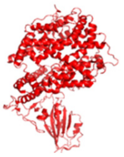
