# Supplementary material for: Integrative Bioinformatics Approaches Indicate a Particular Pattern of Some SARS-CoV-2 and Non-SARS-CoV-2 Proteins
Source: Vaccines (Basel). 2022 Dec 23;11(1):38. doi: 10.3390/vaccines11010038 (PMC9864461; doi:10.3390/vaccines11010038)
Supplement: Supplementary file 1 [file vaccines-11-00038-s001.zip › Table S6.pdf]

Table S6. The generated alphabets and the protein alphabets (Image obtained from PDB) were used in the image comparison study of "COVID-19".

Sl. No.	Pattern of the 3D structure of the protein as Alphabet/Numbers/ Characters used in this study	Created image of alphabets	Protein alphabets (Image obtained from PDB)
1.	C	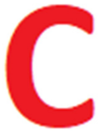	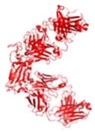
2.	O	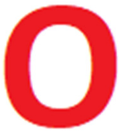	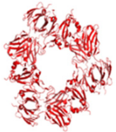
3.	V	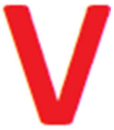	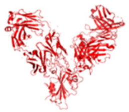
4.	I	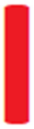	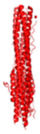
5.	D	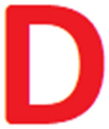	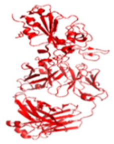
6.	- (Hyphen)	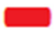	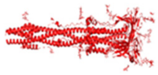
7.	1	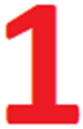	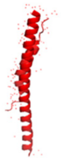
8.	9	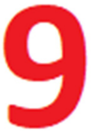	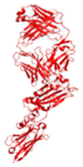
