# Supplementary material for: Integrative Bioinformatics Approaches Indicate a Particular Pattern of Some SARS-CoV-2 and Non-SARS-CoV-2 Proteins
Source: Vaccines (Basel). 2022 Dec 23;11(1):38. doi: 10.3390/vaccines11010038 (PMC9864461; doi:10.3390/vaccines11010038)
Supplement: Supplementary file 1 [file vaccines-11-00038-s001.zip › Table S7.pdf]

Table S7. The generated alphabets and the protein alphabets (image obtained from PDB) were used in the image comparison study using the protein having antibodies/immunological or vaccine-associated roles.

Sl. No.	Pattern of the 3D structure of the protein as alphabet/numbers/ characters used in this study	Created image of alphabets	Protein alphabets (image obtained from PDB)
1.	A	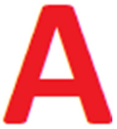	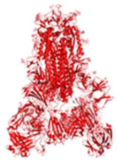
2.	A	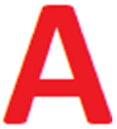	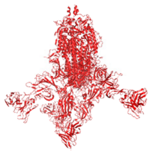
3.	D	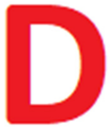	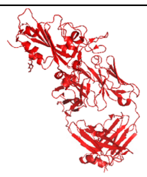
4.	Y	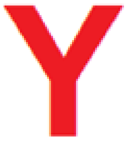	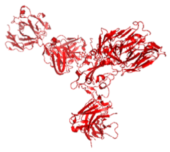
